# Natural‐based antioxidants in cosmeceuticals: Extraction, bioavailability and skin ageing applications

**DOI:** 10.1111/ics.70039

**Published:** 2025-10-24

**Authors:** Hossein Omidian, Arnavaz Akhzarmehr, Charise Dallazem Bertol

**Affiliations:** ^1^ Barry and Judy Silverman College of Pharmacy Nova Southeastern University Fort Lauderdale Florida USA; ^2^ Universidade de Passo Fundo/University of Passo Fundo Passo Fundo RS Brazil

**Keywords:** advanced delivery systems, antiaging skincare, antioxidants, leaf extracts, sustainable cosmeceuticals

## Abstract

This review offers an in‐depth exploration of the bioactivities, extraction techniques, formulation approaches and practical uses of naturally derived antioxidants in anti‐ageing skincare. A critical analysis of the literature was performed. Extracts from leaves, aerial parts, seeds, peels, fruits and barks exhibit potent antioxidant, anti‐inflammatory, photoprotective and anti‐tyrosinase activities. Natural‐based antioxidants exhibit a wide range of bioactivities as neutralizing free radicals through mechanisms such as metal chelation and activation of cellular antioxidant pathways (e.g. Nrf2/ARE) and anti‐inflammatory effects by modulating cytokines like TNF‐α and IL‐6 and promoting wound healing by stimulating collagen synthesis and bioactive compound production. Extracts from Mucuna species, *Magnolia officinalis* and *Arbutus unedo*, for instance, demonstrate anti‐ageing efficacy by inhibiting enzymes such as collagenase, elastase and MMPs. Certain fruit and seed extracts provide photoprotection with high SPF values, while others—such as mushroom extracts and essential oils—display potent antimicrobial activity. Their bioactivity is often enhanced through fermentation processes, innovative delivery systems like liposomes, niosomes and polymeric micelles, which improve stability, bioavailability and topical effectiveness. Extraction methods for natural antioxidants—including aqueous, hydroalcoholic, ultrasound‐assisted (UAE), fermentation‐assisted and alternative solvent (NaDES) techniques—are crucial for recovering and stabilizing bioactive compounds. Emerging green technologies such as supercritical CO_2_ extraction (SC‐CO_2_), subcritical water extraction (SWE), supramolecular solvents (SUPRAS) and cloud point extraction (CPE) offer sustainable and selective recovery of bioactives with reduced environmental impact. These bioactives address oxidative stress, UV damage and dermal ageing, offering multifunctional applications in cosmeceuticals, pharmaceuticals and nutraceuticals. However, challenges such as photostability, inconsistent bioavailability and regulatory hurdles persist. Future research focusing on synergistic formulations, clinical validation and microbiome‐friendly antioxidants will drive their advancement in next‐generation sustainable skincare.

## Graphical abstract

Natural antioxidants from plants, fruits, seeds and fungi combat ageing by neutralizing free radicals, boosting collagen and protecting against UV damage. Enhanced by green extraction and smart delivery systems, they offer powerful anti‐inflammatory and antimicrobial benefits. These bioactives are reshaping skincare—merging nature, science and sustainability in next‐generation therapeutic formulations.

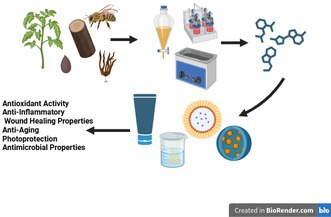

## Résumé

Cette étude propose une exploration approfondie des bioactivités, des techniques d'extraction, des approches de formulation et des utilisations pratiques des antioxydants d'origine naturelle dans les soins anti‐âge pour la peau. Une analyse critique de la littérature a été réalisée. Les extraits de feuilles, de parties aériennes, de graines, de zestes, de fruits et d'écorces présentent de puissantes activités antioxydantes, anti‐inflammatoires, photoprotectrices et anti‐tyrosinase. Les antioxydants d'origine naturelle présentent un large éventail de bioactivités, comme la neutralisation des radicaux libres par des mécanismes tels que la chélation des métaux et l'activation des voies antioxydantes cellulaires (par exemple, Nrf2/ARE), des effets anti‐inflammatoires par la modulation de cytokines telles que le TNF‐α et l'IL‐6, et la promotion de la cicatrisation des plaies en stimulant la synthèse de collagène et la production de composés bioactifs. Les extraits de Mucuna, de Magnolia officinalis et d'Arbutus unedo, par exemple, démontrent une efficacité anti‐âge en inhibant des enzymes telles que la collagénase, l'élastase et les MMP. Certains extraits de fruits et de graines offrent une photoprotection avec des indices de protection solaire élevés, tandis que d'autres, tels que les extraits de champignons et les huiles essentielles, présentent une puissante activité antimicrobienne. Leur bioactivité est souvent renforcée par un processus de fermentation et des systèmes d'administration innovants tels que les liposomes, les niosomes et les micelles polymères, qui améliorent la stabilité, la biodisponibilité et l'efficacité topique. Les méthodes d'extraction des antioxydants naturels, notamment les techniques aqueuses, hydroalcooliques, assistées par ultrasons (UAE), assistées par fermentation et à solvants alternatifs (NaDES), sont essentielles pour récupérer et stabiliser les composés bioactifs. Les nouvelles technologies vertes telles que l'extraction au CO₂ supercritique (SC‐CO₂), l'extraction à l'eau subcritique (SWE), les solvants supramoléculaires (SUPRAS) et l'extraction au point de trouble (CPE) permettent une récupération durable et sélective des composés bioactifs avec un impact environnemental réduit. Ces composés bioactifs combattent le stress oxydatif, les dommages causés par les UV et le vieillissement cutané, offrant des applications multifonctionnelles dans les cosméceutiques, les produits pharmaceutiques et les nutraceutiques. Cependant, des défis tels que la photostabilité, la biodisponibilité inégale et les obstacles réglementaires persistent. Les recherches futures axées sur les formulations synergiques, la validation clinique et les antioxydants respectueux du microbiome favoriseront leur progrès dans les soins de la peau durables de nouvelle génération.

## INTRODUCTION

The growing demand for effective and sustainable anti‐ageing solutions has driven extensive research into natural‐based antioxidants, with plants, algae, fungal and bee‐derived compounds emerging as potent bioactive agents. These naturally occurring antioxidants play a crucial role in counteracting oxidative stress, a key contributor to ageing, inflammation and degenerative skin conditions. Oxidative stress arises from an imbalance between reactive oxygen species (ROS) and the skin’s endogenous antioxidant defences, leading to structural protein degradation, inflammation and DNA damage, which accelerate skin ageing. The visible manifestations include wrinkles, loss of elasticity and hyperpigmentation, making antioxidant‐based interventions crucial for maintaining skin health [1, 2].

Natural‐based antioxidants span multiple biological sources, including plant extracts, algae‐derived phlorotannins, mushroom‐based bioactives and bee‐derived compounds like propolis and honey. These compounds exhibit a wide spectrum of therapeutic properties, including free radical scavenging, metal chelation, enzyme inhibition and modulation of cellular antioxidant pathways. Notably, polyphenols, flavonoids, carotenoids and alkaloids present in these natural sources contribute to collagen preservation, elastin integrity, anti‐inflammatory activity and enhanced cellular repair. Their cosmeceutical applications extend to skincare formulations targeting oxidative stress reduction, wrinkle prevention, skin hydration, UV protection and microbiome balance [3, 4, 5, 6].

Innovations in bioactive extraction methods have significantly improved the yield, purity and efficacy of natural antioxidants. Techniques such as ultrasound‐assisted extraction (UAE), hydroalcoholic extraction, solvent‐free natural deep eutectic solvents (NaDES) and fermentation‐assisted extraction have enhanced the bioavailability and sustainability of these compounds. Additionally, fermentation techniques have been employed to amplify the bioactivity of botanical and fungal extracts, increasing their phenolic content and antioxidant capacity [6, 7, 8].

Parallel to these advancements, innovative formulation technologies have improved stability, penetration and controlled release of natural antioxidants in cosmeceutical applications. Nanoformulations such as liposomes, ethosomes, niosomes, magnetoliposomes, nanoemulsions and nanostructured lipid carriers (NLCs) offer enhanced dermal absorption and prolonged activity, overcoming traditional limitations of natural antioxidants' instability and low solubility. These technologies have enabled the development of next‐generation antioxidant skincare formulations that provide sustained protection against environmental stressors, including UV radiation and pollution‐induced oxidative damage [9, 10, 11].

The emphasis on eco‐friendly, renewable and biodegradable ingredients has further bolstered the integration of natural antioxidants in skincare. Many agricultural by‐products and marine biomass sources—such as red grape pomace, olive leaves, seaweed extracts and coffee silverskin—are being repurposed to reduce waste and align with sustainable, circular economy practices. Additionally, natural antioxidants derived from honey and propolis have demonstrated wound healing, antimicrobial and antioxidant benefits, reinforcing their role in dermatological applications [6, 12, 13].

This manuscript provides a comprehensive analysis of bioactivity, extraction methods, formulation strategies and applications of natural‐based antioxidants in anti‐ageing skincare. By evaluating their mechanisms of action, technological innovations, clinical validation and sustainability potential, this work highlights the transformative role of botanical, algae‐derived, fungal and bee‐based antioxidants in next‐generation cosmeceuticals and dermatological treatments. Furthermore, it discusses challenges and future directions, addressing key issues such as bioavailability, regulatory hurdles, standardization and the integration of microbiome‐friendly antioxidants to shape the future of sustainable, effective and science‐backed skincare solutions. This study aims to bridge the gap between research and commercial application, offering insights into how natural antioxidants can rival or surpass synthetic compounds in effectiveness, safety and environmental impact.

## NATURAL‐BASED ANTIOXIDANTS

Natural‐based antioxidants play a crucial role in counteracting oxidative stress, a key factor in ageing, inflammation and degenerative diseases. These bioactive compounds, derived from plants, algae, fungi and bee products, act by neutralizing free radicals, chelating metals and modulating antioxidant enzyme activity. Their applications extend across skincare, pharmaceuticals and nutraceuticals, providing effective solutions for oxidative damage prevention and treatment.

### Plant‐derived antioxidants

Plants serve as rich reservoirs of antioxidants, primarily polyphenols, flavonoids and alkaloids. These compounds help counter oxidative stress and ageing by scavenging free radicals, chelating metals and inhibiting enzymes associated with oxidative damage.

#### Whole plant extracts

Whole plant extracts provide a synergistic combination of phytochemicals that enhance antioxidant efficacy. For instance, *Jasminum sambac* cell extract (JasHEx) has been shown to reduce reactive oxygen species (*ROS*) in keratinocytes under oxidative stress, inhibit the formation of advanced glycation end‐products (*AGEs*) and boost collagen type I production. These effects, linked to radical scavenging, metal chelation and activation of the *Nrf2/ARE* pathway, make JasHEx a valuable ingredient in anti‐ageing skincare [2].

Similarly, *Helichrysum italicum* extracts, obtained via hydroxypropyl‐β‐cyclodextrin‐assisted extraction, demonstrated potent *antioxidant* properties. Their efficacy was confirmed through *DPPH radical scavenging, reducing power* and *UV absorption* assays. These extracts also inhibited key enzymes such as *hyaluronidase, tyrosinase* and *lipoxygenase*, further supporting their potential in cosmeceuticals [14].

To enhance the transdermal delivery of *apigenin*, a poorly water‐soluble bioactive compound, researchers developed a novel niosome‐based gel (*AN‐NS2G4*). The optimized formulation achieved an entrapment efficiency of 86.19%, *antioxidant* activity of 90.72%, and improved drug release compared to conventional formulations. In vivo studies further revealed significant anti‐inflammatory effects, highlighting its therapeutic potential in skincare [15].

Additionally, an analysis of 16 herbal extracts identified *Stevia rebaudiana* as the richest in *phenolic* and *flavonoid* content, while *Rosa damascena* and *Phyllanthus emblica* exhibited strong *antioxidant* and moderate *anti‐tyrosinase* activity, reinforcing their suitability for skincare applications [16]. A broader evaluation of 92 herbal extracts and *propolis* pinpointed *Lamium purpureum, Garcinia mangostana, Pistacia vera, Punica granatum* and *Vitis vinifera* as the most potent *antioxidants*. These extracts effectively inhibited *elastase, collagenase* and *tyrosinase*—enzymes crucial in skin ageing [17].

#### Aerial parts

Extracts from the aerial parts of *Echinacea purpurea*, optimized using ultrasound‐assisted glycerol extraction, have demonstrated high *caffeic acid* content. These extracts exhibit strong radical scavenging, metal‐chelating and enzyme‐inhibitory activities, indicating significant anti‐ageing potential and suitability for cosmeceutical applications [18].

Similarly, *Heliotropium bacciferum* extract, incorporated into a topical ointment, showed strong antioxidant activity. In Wistar rats, it enhanced wound contraction, increased *glutathione* levels and boosted *catalase* and *superoxide dismutase* activity. Histological studies further confirmed improved collagen deposition and epidermal regeneration, supporting its potential in wound healing [19].

#### Leaves

Leaf‐derived antioxidants have also proven highly effective. *Ilex paraguariensis* extracts encapsulated in polymeric nanoparticles exhibited improved antioxidant stability and resistance to premature degradation, ensuring prolonged activity on the skin surface [20].

Additionally, polyphenol‐rich extracts from *Vitis vinifera* and *Vitis labrusca* grape leaves displayed strong free radical scavenging properties and UV‐protective effects, making them particularly valuable for phytocosmetic formulations [21].

Meanwhile, *Arbutus unedo* extracts obtained via water reflux extraction contained high levels of *polyphenols, flavonoids* and *tannins*. When formulated into nanoemulsions, these extracts demonstrated strong antioxidant and anti‐tyrosinase activity, highlighting their potential for skin‐whitening and protection against oxidative stress [22].

#### Fruits and peels

Fruits and peels also serve as rich sources of antioxidants. *Polyphenols* extracted from grape pomace using natural deep eutectic solvents (*NaDESs*) exhibited intracellular antioxidant activity, protecting human keratinocytes from oxidative stress while reducing IL‐8 levels, a marker of inflammation [6]. Similarly, *Hylocereus polyrhizus* (*dragon fruit*) peel extract displayed strong antioxidant activity and substantial UV absorbance. These properties suggest its potential as a natural sunscreen ingredient with photoprotective effects [23].

#### Bark and seeds

Bark‐ and seed‐derived antioxidants have demonstrated significant efficacy. Fermentation of *Magnolia officinalis* bark with *Aspergillus niger* increased its total *phenolic* content by 3.52 times, enhancing its DPPH radical scavenging capacity and antibacterial activity against methicillin‐resistant *Staphylococcus aureus* (*MRSA*). These findings underscore its potential as an anti‐ageing agent [13]. In a separate study, seeds from *Mucuna* species—particularly *Mucuna pruriens* and *Mucuna gigantea*—exhibited the highest antioxidant activity. They also significantly inhibited *collagenase, elastase* and *hyaluronidase*, enzymes involved in skin ageing, suggesting their potential applications in anti‐ageing skincare formulations [24].

#### Specialized plant compounds

Plant‐derived antioxidants also include specific bioactive compounds that enhance antioxidant efficacy. Essential oils from oregano, basil and lemon balm were formulated into nanoemulsions, with oregano‐based formulations demonstrating the highest stability and prolonged antioxidant activity [8]. Polyphenol‐based antioxidants, such as *resveratrol*, have also been optimized for skincare applications. *Resveratrol*‐loaded liposomes and niosomes exhibited superior skin retention and penetration, maximizing their protective effects against oxidative damage [25]. Among alkaloid‐based antioxidants, *berberine*‐loaded oleosomes provided sustained release, enhanced bioavailability and significant therapeutic potential in treating oxidative stress‐related skin conditions [26].

### Algae‐derived antioxidants

Algae are rich sources of antioxidants, including *phlorotannins, carotenoids* and *sulfated polysaccharides*.

Extracts from brown algae, such as *Fucus vesiculosus*, contain high levels of *phlorotannins*, which have demonstrated strong antioxidant properties [10]. Similarly, *Agarum cribrosum* extracts significantly inhibited *collagenase, elastase* and *tyrosinase*—enzymes associated with skin ageing—supporting their use in anti‐ageing skincare [27]. Cyanobacteria (blue–green algae) also exhibit unique antioxidant properties. Extracts from *Cyanobium, Leptothoe* and *Synechococcales* inhibited oxidative stress‐related enzymes, highlighting their potential for skincare formulations [28]. Additionally, red macroalgae, such as *Sargassum thunbergii*, demonstrated strong radical scavenging activity, increased intracellular *antioxidant* enzyme levels and inhibited *matrix metalloproteinases*, which contribute to skin ageing [29].

### Bee‐derived antioxidants

Bee‐derived antioxidants, particularly *propolis* and *honey*, offer strong free radical scavenging, antibacterial and wound‐healing effects. Brazilian red *propolis* (*BRP*) has been shown to accelerate wound healing by increasing *antioxidant* enzyme activity and enhancing collagen synthesis in a rat skin excision model [30]. Similarly, Western Australian and *Manuka honey*‐based topical formulations retained their *antioxidant* and antibacterial properties, making them effective candidates for therapeutic skincare [31].

### Other natural‐based antioxidants

Mushroom‐derived *antioxidants* have also gained attention for their potent bioactivity. Hydroethanolic extracts from *Agaricus bisporus, Boletus edulis* and *Lentinula edodes* exhibited strong *antioxidant* and antibacterial properties, supporting their potential use in cosmeceutical applications [7]. Additionally, food by‐product‐derived *antioxidants* provide sustainable alternatives. *Coffee silverskin* extracts, when incorporated into skincare formulations, improved skin hydration and retained *antioxidant* properties over time [32]. Similarly, extracts from *palm kernel cake* displayed strong metal‐chelating and *antioxidant* activity, further reinforcing their suitability for cosmetic applications [33].

Table 1 outlines the diverse sources of antioxidants, categorized into algae, fungi, bee products, food by‐products and various plant parts. Plant‐based antioxidants dominate, with leaves, polyphenols, flavonoids and essential oils being the most represented categories. Algae‐derived antioxidants, such as phlorotannins and cyanobacteria extracts, highlight marine sources' growing role in antioxidant applications. Bee‐derived products (propolis) and food by‐products (coffee silverskin, palm kernel extract) showcase sustainable antioxidant sources. Whole plant and mixed plant parts demonstrate versatility in bioactive compound extraction. Flavonoid and polyphenol‐rich extracts, including quercetin, rutin, resveratrol and curcumin, are widely studied for their potent antioxidant and anti‐inflammatory properties. The table reflects a broad spectrum of natural‐based antioxidants with applications in cosmetics, pharmaceuticals and nutraceuticals.

**TABLE 1:** Diverse sources of natural antioxidants: classification by origin and bioactive compounds.

Source type	Examples	References
Algae	*Fucus vesiculosus* extract, red marine macroalgae extracts, *Agarum cribrosum* phlorotannins, *Sargassum thunbergii* ethanolic extract, Cyanobacteria extracts, *Arthrospira platensis* extract	[10, 27, 28, 29, 34, 35]
Fungi	Edible mushroom extracts	[7]
Bee products	Propolis extract, Brazilian Red Propolis	[30, 36]
Food by‐products	Coffee silverskin (*Coffea* sp.) and *Medicago sativa* extracts, Palm kernel cake (PKC) protein extract	[32, 33]
Natural products	Honey‐loaded topical formulations	[31]
Halophyte	*Mesembryanthemum nodiflorum* extract	[37]
Plant—aerial parts	*Echinacea purpurea* glycerolic extract, *Bergia ammannioides* extract, *Leonurus sibiricus* (Motherwort) extract, *Baccharis incarum* extract, *Heliotropium bacciferum* extract, *Bunium alpinum* extract, *Oxybaphus nyctagineus* extract	[18, 19, 38, 39, 40, 41, 42]
Plant—alkaloid	Berberine‐loaded gel‐core oleosomes	[26]
Plant—bark	*Magnolia officinalis* bark extract (fermented by *Aspergillus niger*), *Terminalia arjuna* extract	[13, 43]
Plant—berry	*Vaccinium myrtillus* (blueberry) polyphenols, *Rubus fruticosus* (blackberry) & *Rubus idaeus* (raspberry) extracts	[44, 45]
Plant—bioactive Compound	Carotenoids (topical & systemic), Hesperetin‐loaded proposomal gel, Eucalyptol (topical ointment), various antioxidants (fisetin, retinol, cyanidin, hesperetin, caffeic acid, luteolin, rutin, etc.)	[46, 47, 48, 49]
Plant—Essential oils	Essential oils (Basil, Lemon Balm, Oregano), Geranium and Calendula essential oils, essential oils (Menthol, Thymol) & polyphenolic antioxidants	[8, 50, 51]
Plant—Flavonoid	Rutin‐loaded ethosomes, Quercetin‐loaded mixed micelles, Quercetin‐loaded liposomes & chitosomes, Quercetin‐loaded magnetoliposomes (QMLs), Quercetin‐loaded polyoxazoline lipid nanocapsules (LNC POx), Apigenin (Flavonoid)	[9, 11, 52, 53, 54]
Plant—Flower	*Calendula officinalis* extract, *Calendula officinalis* cream, *Epilobium angustifolium* extract in bacterial cellulose membrane	[55, 56, 57]
Plant—Fruit	*Malus sylvestris* (wild apple) fruit extract, high‐molecular‐weight polysaccharides from *Diospyros kaki* (Persimmon), lacto‐fermented microencapsulated guava juice powder, *Hylocereus polyrhizus* (red dragon fruit) peel extract	[23, 58, 59, 60]
Plant—Leaf	*Castanea sativa* leaf extract, *Moringa oleifera* leaf extract, *Ximenia americana var. caffra* leaf extract, *Vitis vinifera* & *Vitis labrusca* grape leaf polyphenols, *Arbutus unedo* phenolic extract, *Dianella ensifolia* extract, *Calendula officinalis* leaf extract, *Cecropia pachystachya* extract, *Medicago lupulina* (Black Medick) extract	[1, 3, 5, 21, 22, 61, 62, 63, 64]
Plant—Meristematic	*Buddleia davidii* extract (Verbascoside & VPP)	[65]
Plant—Mixed parts	*Eleocarpus serratus*, *Curcuma aromatica*, *Artocarpus altilis*, *Artocarpus nobilis* extracts, *Pandanus amaryllifolius* leaf & root extract	[66, 67]
Plant—Nut	Chestnut inner shell extract (fermented by *Monascus kaoliang*)	[12]
Plant—Peel	*Vitis vinifera* (Benitaka Grape) peel extract, *Hylocereus polyrhizus* (red dragon fruit) peel extract	[23, 68]
Plant—Polyphenols	Caffeic & ferulic acid‐loaded lipid vesicular gels, curcumin‐loaded niosomes, resveratrol‐loaded ultradeformable vesicular cream, Trans‐resveratrol microemulsions (RO‐ME, JO‐ME, LO‐ME), resveratrol nanoemulsions with terpenes, resveratrol‐loaded liposomes & niosomes, curcumin analogue‐loaded nanoparticle (CA‐NP) gel, green tea polyphenols (GTP), ascorbyl tetraisopalmitate, Coenzyme Q_10_, resveratrol, resveratrol, baicalin, vitamin E (Topical Blend), gallic acid, carvacrol and magnolol	[25, 69, 70, 71, 72, 73, 74, 75, 76, 77, 78]
Plant—Seed	Mucuna seed extracts (*M. gigantea*, *M. interrupta*, *M. monosperma*, *M. pruriens*), *Hydnocarpus anthelmintica* extract, Sesamol (*Sesamum indicum* extract)	[24, 79, 80]
Plant—Stem	*Tinospora cordifolia* nanoparticles	[81]
Plant—Whole Plant	*Ajuga spectabilis* Nakai extract, *Helichrysum italicum* extract, *Crataegus monogyna* (Hawthorn), Various phenolic compounds from *Philadelphus coronarius* (Sweet mock‐orange), Eugenol, tannic acid, rutin from *Pimenta pseudocaryophyllus*, *Ocimum basilicum* and *Trifolium pratense* extracts, *Stevia rebaudiana*, *Rosa damascena*, *Phyllanthus emblica*, *Echinacea purpurea*, *Morus alba* extracts, *Lamium purpureum*, *Garcinia mangostana*, *Pistacia vera*, *Punica granatum*, *Vitis vinifera* extracts	[4, 14, 16, 17, 82, 83, 84, 85]

## BIOACTIVITY OF NATURAL‐BASED ANTIOXIDANTS

Natural‐based antioxidants exhibit diverse bioactivities, including antioxidant, anti‐inflammatory, anti‐ageing, wound‐healing, antimicrobial and UV‐protective properties. These bioactivities contribute significantly to their applications in pharmaceutical, cosmeceutical and dermatological fields. Below, we summarize key bioactivities observed in various natural antioxidant extracts and formulations.

### Antioxidant activity and free radical scavenging

Natural antioxidants effectively neutralize free radicals through scavenging mechanisms (DPPH, ABTS, FRAP assays), metal chelation and oxidative stress inhibition. *Jasminum sambac* extract enhances cellular antioxidant response via the Nrf2/ARE pathway, while *Malus sylvestris* fruit extract, rich in polyphenols, demonstrates high radical scavenging activity (DPPH: 8.12–78.43% RSC; FRAP: 2.13–7.65 mM Fe^2+^) [2, 58]. *Ximenia americana var. caffra* leaf extract exhibits dose‐dependent ROS reduction in oxidative stress‐induced *C. elegans*, significantly improving survival rates (68.9% vs. 24.8% in control) [5]. Grape pomace polyphenols show intracellular antioxidant activity, reducing IL‐8 release and improving cell viability (IC_50_: 0.15 ± 0.02 μg/mL) [6]. Encapsulation enhances antioxidant efficiency: Quercetin‐loaded liposomes and chitosomes exhibit superior antioxidant activity due to chitosan synergy, allowing controlled release and improved skin retention [52]. Similarly, quercetin‐loaded mixed micelles remain stable for 28 days while retaining antioxidant potency and reducing cytotoxicity [11].

### Anti‐inflammatory and wound‐healing properties

Natural antioxidants modulate inflammation by inhibiting TNF‐α, IL‐6, IL‐1β, COX‐2 and iNOS. *Bergia ammannioides* extract reduces inflammation by 64.5%, comparable to Voltaren® (a standard anti‐inflammatory drug), while enhancing collagen content for wound healing [38]. *Echinacea purpurea* glycerolic extract inhibits collagenase, elastase and hyaluronidase, reinforcing its skin repair and anti‐ageing potential [18]. *Cecropia pachystachya* extract promotes wound healing by increasing bioactive compounds such as orientin (66.5 μg/mg), isoorientin (118.8 μg/mg) and chlorogenic acid (5.4 μg/mg), all contributing to its anti‐inflammatory and antioxidant effects [63]. Berberine‐loaded gel‐core oleosomes improve skin retention and histological confirmation of efficacy, outperforming free berberine in anti‐inflammatory applications [26].

### Anti‐ageing and collagen protection

By inhibiting collagen‐degrading enzymes (collagenase, elastase, MMPs), natural antioxidants help maintain skin firmness. *Magnolia officinalis* bark extract, when fermented by *Aspergillus niger*, enhances inhibition of MMP‐1, MMP‐2, collagenase and elastase, reducing wrinkles and signs of ageing [13]. Similarly, Mucuna species (*M. gigantea, M. pruriens, M. monosperma*) demonstrate strong collagenase, elastase and hyaluronidase inhibition, making them promising anti‐ageing agents [24]. *Arbutus unedo* extract combines anti‐inflammatory and anti‐tyrosinase activity, with its high hyperoside content supporting cosmeceutical applications [22]. Advanced formulations such as quercetin‐loaded magnetoliposomes (QMLs) enhance bioavailability and retain strong antioxidant properties, improving anti‐ageing effects [53]. Likewise, resveratrol‐loaded nanoemulsions containing terpenes (eugenol, limonene) penetrate deeper into the epidermis, further boosting anti‐ageing benefits [73].

### UV protection and photoprotection

Many plant‐derived antioxidants provide UV protection by absorbing harmful radiation and neutralizing free radicals. Blackberry and raspberry extracts demonstrate high SPF values of 54.57 and 37.32, respectively, while *Vitis vinifera* (*Benitaka grape* peel extract) offers UVA/UVB protection (SPF: 18.56; critical wavelength: 318.0 ± 0.1 nm) [45, 68]. Synergistic formulations amplify UV protection. A topical blend of resveratrol, niacinamide and GHK peptide improves skin elasticity, reduces wrinkles and enhances radiance after 12 weeks [76]. Similarly, trans‐resveratrol microemulsions increase solubility and photostability, improving UVB protection and free radical scavenging activity [72].

### Antimicrobial properties

Certain natural antioxidants exhibit potent antimicrobial effects against pathogenic bacteria, often linked to polyphenols, flavonoids or essential oils. Edible mushroom extracts (*Boletus edulis, Agaricus bisporus*) inhibit bacterial growth, with MIC values ranging from 5 to 20 mg/mL for Gram‐positive bacteria and 10–20 mg/mL for Gram‐negative bacteria [7]. Fermented extracts show enhanced antimicrobial efficacy. Chestnut inner shell extract, fermented by *Monascus kaoliang*, demonstrates increased bioactivity after 3 days of fermentation [12]. Brazilian red propolis (BRP) boosts enzymatic defences (SOD, MPO, GSH, GR), contributing to its wound‐healing and antibacterial potential [30]. Among essential oils, geranium and calendula essential oils provide antioxidant activity and mild sun protection (SPF: 6.45 and 8.36, respectively) [50]. Meanwhile, oregano essential oil nanoemulsions (OR‐NE) retain their antioxidant activity for over 6 months, offering stable antimicrobial benefits [8].

Table 2 highlights key bioactivities of natural antioxidants, revealing patterns in their therapeutic and cosmeceutical potential. Most extracts exhibit antioxidant and free radical scavenging properties, often enhanced through nanoencapsulation or fermentation for improved bioavailability (e.g. rutin‐loaded ethosomes, berberine‐loaded oleosomes). Many also show anti‐inflammatory effects, inhibiting TNF‐α, IL‐6 and COX‐2 (e.g. *Echinacea purpurea*, *Bergia ammannioides*). Wound‐healing and anti‐ageing benefits correlate with collagenase and elastase inhibition (e.g. *Mucuna seed extracts*). UV protection is significant in high‐SPF extracts (e.g. *Blackberry & Raspberry*), while several possess antimicrobial properties beneficial for skin health (e.g. *Edible mushrooms*). These insights support their dermatological and pharmaceutical applications.

**TABLE 2:** Bioactivity of natural‐based antioxidants.

Bioactivity	Description	Key natural extracts	References
Antioxidant activity	Many plant‐based extracts demonstrate strong antioxidant properties via DPPH, FRAP and ABTS assays, reducing oxidative stress	*Jasminum sambac*, *Malus sylvestris*, Grape pomace polyphenols	[2, 6, 58]
Free Radical Scavenging	Scavenging of free radicals and metal chelation improve cellular protection; dose‐dependent effects observed in multiple studies	*Ximenia americana*, Quercetin‐loaded mixed micelles, *Castanea sativa*	[1, 5, 11]
Enhanced Stability & Bioavailability	Fermentation, encapsulation and nanoformulations significantly enhance stability, bioavailability and efficacy of antioxidants	Rutin‐loaded ethosomes, *Magnolia officinalis* (fermented), Berberine‐loaded oleosomes	[9, 13, 26]
Anti‐Inflammatory Effects	Reduces inflammatory markers (TNF‐α, IL‐6, IL‐1β, COX‐2, iNOS); shown to suppress cytokine production and oxidative damage	*Echinacea purpurea*, *Bergia ammannioides*, *Cecropia pachystachya*	[18, 38, 63]
Wound Healing	Promotes collagen synthesis, reduces inflammation and enhances fibroblast viability for improved wound repair	Brazilian red propolis, *Helichrysum italicum*, *Dianella ensifolia*	[14, 30, 61]
Anti‐Aging & Collagen Protection	Inhibits collagenase, elastase and MMPs; prevents skin ageing by improving skin elasticity, firmness, and reducing wrinkles	Mucuna seed extracts, *Magnolia officinalis* (fermented), *Arbutus unedo*	[13, 22, 24]
UV Protection & Photoprotection	Provides SPF protection, prevents UV‐induced oxidative stress and improves photostability for long‐term skincare applications	Blackberry & Raspberry, *Vitis vinifera* peel extract, Trans‐resveratrol nanoemulsions	[45, 68, 73]
Antimicrobial Activity	Demonstrates antibacterial and antifungal properties; active against Gram‐positive and Gram‐negative bacteria, improving skin health	Edible mushrooms, Geranium & Calendula essential oils, Oregano EO nanoemulsion	[7, 8, 50]

Table 3 outlines highly effective antioxidants with proven stability, bioavailability and diverse therapeutic applications. Flavonoid‐rich extracts like *Moringa oleifera*, Grape pomace polyphenols and Quercetin‐loaded mixed micelles show strong antioxidant activity, inflammation reduction and long‐term stability. Encapsulated antioxidants (e.g. Resveratrol, Curcumin, EGCG and Ilex paraguariensis extract) demonstrate enhanced skin penetration, prolonged antioxidant action and potential for anti‐ageing and cancer prevention. Propolis and Cyanobacteria extracts highlight natural sources with UV protection, antimicrobial properties and enzymatic inhibition. Carotenoids and *Calendula officinalis* extracts are recognized for skin health benefits and long‐lasting antioxidant effects. This table presents a comprehensive selection of bioactive compounds optimized for medical, cosmetic and nutraceutical applications.

**TABLE 3:** Key antioxidants with high stability, bioavailability and therapeutic potential.

Antioxidant	General features	References
*Moringa oleifera* leaf extract	Flavonoid‐rich; inhibits lipid peroxidation; maintains skin pH and hydration. Hydroalcoholic fraction; contains flavonoids and phenolic acids; inhibits lipid peroxidation; maintains skin pH and hydration	[3]
Grape pomace polyphenols	High malvidin content; strong intracellular antioxidant activity; reduces inflammation. Extracted using NaDES solvents; Malvidin content (51–56 μg/mL); Intracellular antioxidant activity (IC_50_ = 0.15 ± 0.02 μg/mL); Significantly decreased IL‐8 release (*p* < 0.001)	[6]
Edible mushroom extracts	Inhibits hyaluronidase; rich in p‐Hydroxybenzoic and Gallic acids; antimicrobial effects. p‐Hydroxybenzoic acid (0.010–2.554 μg/g DW); Gallic acid (0.032–0.112 μg/g DW); B. edulis extract MIC = 5–20 mg/mL (Gram‐positive), 10–20 mg/mL (Gram‐negative); A. bisporus extract (10 mg/mL) inhibited hyaluronidase by 74.4 ± 7.5%	[7]
Quercetin‐loaded mixed micelles	Encapsulation efficiency of 94%; stable for 28 days with unchanged activity; strong free radical scavenging. Encapsulation efficiency: 94 ± 4%; Drug loading: 3.6 ± 0.2%; Nanosized vesicles (~20 nm); Stable for 28 days; Antioxidant activity remained unchanged	[11]
*Calendula officinalis* extract	Contains carotenoids, polyphenols, flavonoids; long‐term antioxidant stability; anti‐inflammatory. Cream with 0.9% Calendula extract had 0.73 ± 0.04 mg/100 g total carotenoids (β‐carotene equivalent); Antioxidant activity due to carotenoids, polyphenols, flavonoids; Stability tests confirmed long‐term effectiveness	[55]
Carotenoids (topical & systemic)	Enhances skin protection; systemic storage up to 5 weeks; improves dermal health. Topical, systemic, and combined administration increased dermal carotenoid levels; Systemic storage lasted up to 5 weeks; Topical lasted ~2 weeks; Combined method most effective	[46]
Resveratrol‐loaded liposomes & niosomes	Stable vesicle formulation enhances resveratrol skin penetration; anti‐aging and anti‐inflammatory. Vesicle size ~200 nm; Peceol niosomes provided high cutaneous accumulation but low transdermal permeation; Stable formulations optimized for topical resveratrol delivery	[25]
Curcumin‐loaded niosomes	Effective against oral cancer cells; systemic and topical applications; strong anti‐inflammatory. Curcumin‐loaded niosomes (16 μg dose) inhibited oral cancer cell growth; Injectable curcumin niosomes significantly prevented severe dysplasia in rats; Effective in both systemic and topical (mouthwash) applications	[70]
Propolis extract	Strong UV protection; inhibits lipid peroxidation; antimicrobial and anti‐inflammatory effects. Strong UV protection (broad‐spectrum UVA/UVB absorption); High antioxidant activity (inhibits lipid peroxidation); Potential sunscreen cosmeceutical ingredient.	[36]
Epigallocatechin‐3‐gallate (EGCG) with co‐antioxidants	Stabilized with alpha‐lipoic acid; maintains high antioxidant activity under sunlight; enhances skin health. EGCG degrades under sunlight (76.9% loss); Alpha‐lipoic acid stabilizes EGCG (12.6% loss, antioxidant activity decrease: 1.4%); Vitamin C also protective (20.4% loss)	[86]
*Ilex paraguariensis* (Yerba mate) extract	Polymeric nanoparticles enhance stability; long‐lasting topical antioxidant effect. Nanoparticles stabilized extract; Polycaprolactone‐based more stable; No ex vivo skin permeation for 12 h; Enhanced topical antioxidant effect	[20]
Cyanobacteria extracts	Rich in carotenoids; strong O_2_ scavenging; high hyaluronidase and collagenase inhibition. Rich in carotenoids (Zeaxanthin: 53.08 μg/mg; Beta‐carotene: 47.89 μg/mg); Strong O_2_ scavenging (IC50 = 63.24 μg/mL); Hyaluronidase inhibition (IC50 = 483.86 μg/mL); Collagenase inhibition (32.88%)	[28]

## EXTRACTION METHODS AND FORMULATION OF NATURAL‐BASED ANTIOXIDANTS

### Aqueous and hydroalcoholic extractions of natural‐based antioxidants

Extraction methods play a crucial role in determining the bioactivity, stability and composition of natural antioxidants, ultimately shaping their applications in health, skincare and pharmaceuticals. Among these, aqueous and hydroalcoholic extractions enhance the recovery of bioactive molecules, producing extracts rich in phenolics, flavonoids and polysaccharides that exhibit antioxidant, anti‐inflammatory and cosmeceutical properties.

Studies have shown that extracts from *Ajuga spectabilis* and *Fucus vesiculosus*, obtained through aqueous and hydroalcoholic methods, exhibit variations in antioxidant and anti‐inflammatory activities. Similarly, hydroethanolic extracts of *Jasminum sambac* and *Moringa oleifera* demonstrate high bioactivity, particularly in lipid peroxidation inhibition and antioxidant potential [2, 3, 4, 10]. Marine macroalgae, such as *Phycocalidia acanthophora* and *Porphyra linearis*, have been found to offer superior collagenase inhibition when extracted in aqueous solutions [34].

In cosmetic applications, *Calendula officinalis* leaf extracts, incorporated into gels and creams, display antioxidant activity but exhibit limited SPF potential. In contrast, hydroethanolic extracts of *Vaccinium myrtillus*, when formulated into ultradeformable liposomes, retain 85% of their antioxidant capacity, enhancing skin penetration [44, 62]. Extracts of *Leonurus sibiricus* and *Heliotropium bacciferum* promote anti‐inflammatory and Wound‐healing properties, improving collagen deposition in topical formulations. Moreover, fractionated polysaccharides from *Diospyros kaki* demonstrate superior antioxidant and anti‐wrinkle effects [19, 39, 59].

Optimization studies on *Sargassum thunbergii* extract (STEE) reveal that an ethanol concentration of 40.2% and a 40‐min extraction time maximize its antioxidant and anti‐inflammatory properties. Meanwhile, Brazilian red propolis (BRP) hydroalcoholic extract has been shown to enhance wound contraction, epithelialization and collagen synthesis while improving SOD and GSH activity [29, 30].

Sequential extraction techniques further reveal that aqueous cyanobacteria extracts are particularly rich in phycobiliproteins, whereas acetone extracts contain carotenoids and phenols, both of which exhibit anti‐ageing potential [28]. In addition, *Pimenta pseudocaryophyllus* ethanolic extracts provide UVB protection and antioxidant benefits in topical formulations. Similarly, hydroalcoholic extracts of *Agaricus bisporus*, *Pleurotus ostreatus* and *Lentinula edodes* preserve their phenolic and ergosterol content, supporting their anti‐inflammatory and anti‐tyrosinase properties in cosmetic creams [84, 87].

Large‐scale screenings have identified *Stevia rebaudiana*, *Rosa damascena* and *Phyllanthus emblica* as potent antioxidant and skin‐whitening agents, with *Morus alba* demonstrating the highest anti‐inflammatory activity [16]. Additionally, *Garcinia mangostana*, *Pistacia vera* and *Punica granatum* exhibit strong enzyme inhibition, reinforcing their potential for cosmeceutical applications [17].

### Ultrasound‐assisted and solvent‐based extractions of natural antioxidants

Ultrasound‐assisted extraction (UAE) is an efficient method for isolating bioactive compounds, improving mass transfer while reducing extraction time, solvent use and energy consumption. Widely applied in cosmeceuticals, nutraceuticals and pharmaceuticals, UAE—along with solvent‐based extractions—enhances antioxidant yield and bioactivity. Additionally, techniques such as natural deep eutectic solvents (NaDES) further contribute to the sustainability and efficiency of these methods.

Hydroethanolic UAE of *Agaricus bisporus*, *Boletus edulis*, *Lentinula edodes* and *Pleurotus ostreatus* mushrooms has been shown to produce high‐phenolic extracts with strong antioxidant and antibacterial properties. Similarly, UAE of *Malus sylvestris* (wild apple) fruit resulted in the highest polyphenol content and antioxidant activity when compared to maceration, percolation and Soxhlet extraction [7, 58]. Furthermore, UAE‐optimized *Echinacea purpurea* glycerol extracts exhibited strong collagenase, elastase and tyrosinase inhibition, confirming their anti‐ageing potential. This technique also improved the extraction efficiency of *Arbutus unedo* compared to conventional methods [18, 22].

In skincare applications, *Pandanus amaryllifolius* extracts, obtained via ethanol and propylene glycol maceration, demonstrated both antioxidant activity and stability in oil‐in‐water emulsions, supporting their suitability for cosmetic formulations. Similarly, *Medicago lupulina* phenols extracted using UAE with glycerol exhibited strong antioxidant, anti‐lipoxygenase and antidiabetic properties [64, 67].

Moreover, phlorotannins from *Agarum cribrosum*, extracted using UAE optimized at 30°C for 5.75 h, displayed potent antioxidant and anti‐ageing effects, with trifuhalol A identified as the key bioactive compound. Additionally, *Hylocereus polyrhizus* (red dragon fruit) peel extract, obtained via ethanolic extraction and analysed using HPLC and UPLC‐QTOF/MS, exhibited high antioxidant activity and strong UV absorption (SPF 35.02 at 1.00 mg/mL), making it a promising natural sunscreen ingredient [23, 27].

### Natural deep eutectic solvents (NaDES) as an alternative to conventional solvents

Natural deep eutectic solvents (NaDES) provide a non‐toxic and biodegradable alternative to conventional organic solvents, making them an ideal choice for sustainable bioactive extraction. These solvents enhance selectivity for phenolic and flavonoid compounds while reducing environmental impact, contributing to greener and more efficient antioxidant recovery.

Research on grape pomace polyphenols has shown that a betaine‐citric acid NaDES mixture offers the highest permeability in Franz diffusion studies, highlighting its potential for improving skin penetration and bioavailability in cosmeceutical applications [6]. Similarly, *Mesembryanthemum nodiflorum* extracts, obtained through supercritical fluid extraction (SFE), have been assessed for anti‐ageing effects in human fibroblasts, confirming their suitability for cosmetic formulations. Among these, ethanol and hexane extracts exhibited the highest antioxidant activity [37].

Additionally, palm kernel cake (PKC) crude protein was extracted at 80°C, followed by ethanol precipitation, producing a protein‐rich extract with strong antioxidant properties. This reinforces its potential use in cosmeceutical formulations [33].

### Fermentation‐assisted extraction of bioactive compounds

Fermentation‐assisted extraction enhances the bioavailability, stability and functionality of bioactive compounds. Through microbial transformation, this technique increases the concentration of phenolic acids, flavonoids and antioxidants, further improving their applications in cosmeceuticals, nutraceuticals and pharmaceuticals. By optimizing the extraction of natural antioxidants, fermentation results in more potent and stable bioactive formulations.

For instance, *Monascus kaoliang*‐fermented chestnut inner shell (CIS) extracts showed an increase in phenolic acids and catechins, leading to enhanced antioxidant activity and cosmeceutical potential. Similarly, *Magnolia officinalis* bark fermentation with *Aspergillus niger* resulted in a 3.52‐fold increase in phenolic content, significantly enhancing anti‐tyrosinase and anti‐ageing effects. This was achieved through the inhibition of collagenase, elastase and MMP‐1/MMP‐2 enzymes, which are associated with skin degradation [12, 13].

Additionally, ethanol extracts of *Bergia ammannioides* displayed diverse bioactivities, with the ethyl acetate fraction (EtFr) exhibiting strong antioxidant properties, while the hexane fraction (HxFr) demonstrated the highest anti‐inflammatory and antibacterial effects. In another study, *Calendula officinalis* creams (10% and 30%) provided antinociceptive, anti‐inflammatory and antioxidant effects, supporting their use in pain relief and skin inflammation treatment [38, 56].

Moreover, lacto‐fermented guava juice, microencapsulated into a powder, exhibited strong antioxidant activity (DPPH IC_50_ = 0.015 mg/mL), and promoted a 31% increase in collagen synthesis in fibroblast cells, confirming its potential for anti‐ageing formulations. Meanwhile, germination of oat (*Avena sativa L*.) over 6 days led to the synthesis of avenanthramides, which effectively inhibited elastase, collagenase and tyrosinase—enzymes linked to wrinkle formation—supporting their role in wrinkle prevention and skin elasticity enhancement [60, 88].

### Nanoformulation and encapsulation techniques for antioxidant delivery

Nanotechnology has revolutionized the delivery of bioactive antioxidants by improving their stability, solubility and skin penetration. Nanoformulated systems—such as ethosomes, liposomes, nanoemulsions and nanocapsules—offer controlled release and prolonged antioxidant effects, making them highly valuable in cosmeceutical, pharmaceutical and nutraceutical applications.

For instance, rutin encapsulated in ethosomes significantly enhanced skin penetration, as confirmed by tape stripping assays. Similarly, quercetin encapsulated in polyoxazoline (POx)‐based micelles achieved a 94% encapsulation efficiency, maintaining nanosized vesicles (~20 nm) while preserving antioxidant activity [9, 11]. Nanoemulsions containing basil, lemon balm and oregano essential oils, stabilized via the phase inversion composition (PIC) method, demonstrated strong antioxidant potential [8]. Additionally, *Ilex paraguariensis* extract encapsulated in polycaprolactone nanoparticles ensured 12‐h topical retention, preventing premature degradation. Meanwhile, quercetin‐loaded liposomes and chitosomes (phosphatidylcholine + chitosan) exhibited high antioxidant stability (78% encapsulation efficiency), supporting efficient skin delivery [20, 52].

Advancements in magnetoliposomes (QMLs), incorporating quercetin and iron oxide nanoparticles, have improved solubility and antioxidant activity, making them promising for Wound‐healing applications [53]. Lipid nanocapsules (LNCs) formulated with polyoxazolines (POx) provided high quercetin retention, effectively preserving antioxidant effects under UVB exposure. Furthermore, caffeic acid and ferulic acid encapsulated in lipid vesicular gels (LVGs) demonstrated controlled release, enhancing antioxidant availability in skin formulations [54, 69].

Berberine‐loaded gel‐core oleosomes (BER‐OS) improved skin penetration and antioxidant potential, showing promise in vitiligo treatment. Additionally, *Tinospora cordifolia* nanoparticles (~182.9 nm) demonstrated cytocompatibility in fibroblast cells, confirming their safety for skincare applications [26, 81]. A resveratrol‐loaded ultradeformable vesicular cream exhibited superior skin deposition and antioxidant preservation compared to conventional creams. Similarly, microemulsions with trans‐resveratrol and essential oils enhanced solubility, photostability and UVB protection [71, 72].

Further innovations include limonene‐based nanoemulsions, which improved resveratrol penetration, while eugenol‐based formulations enhanced retention in deeper skin layers. Curcumin‐loaded slow‐release niosomes also showed strong chemopreventive effects in oral cancer treatment [70, 73]. Additionally, an epigallocatechin‐3‐gallate (EGCG) oil‐in‐water cream, formulated with co‐antioxidants such as vitamin C and alpha‐lipoic acid, improved antioxidant stability under sunlight. A night cream containing resveratrol, baicalin and vitamin E significantly enhanced skin elasticity and photodamage repair [76, 86].

Lastly, sesamol encapsulated in nanostructured lipid carriers (NLCs) achieved over 90% encapsulation efficiency, ensuring prolonged skin absorption. Similarly, phloretin (PHL) incorporated into gliadin–sericin nanostructures (G‐LSS) demonstrated improved UV protection and enhanced skin penetration [80, 89].

### Innovative methods for antioxidant extraction and formulation

The effectiveness of natural antioxidants is greatly influenced by the methods used for their extraction and delivery. Advanced techniques—including direct formulation incorporation, essential oil extraction, biopolymer‐based systems, layer‐by‐layer coatings and systemic antioxidant formulations—help enhance antioxidant potency, improve skin penetration and ensure prolonged therapeutic effects.

For instance, *Castanea sativa* leaf extract integrated into a surfactant‐free formulation maintained prolonged antioxidant activity, following the Higuchi release model. Similarly, *Calendula officinalis* extract incorporated into a hydrophilic cream retained its antioxidant properties, optimizing its long‐term therapeutic applications [1, 55]. Studies have also shown that carotenoid‐based antioxidants exhibit longer retention when administered systemically (up to 5 weeks) compared to topical application, highlighting the impact of delivery routes on antioxidant longevity [46].

Essential oil‐based formulations also demonstrate strong bioactivity. *Pelargonium graveolens* and *Calendula officinalis* essential oils, extracted via hydrodistillation, exhibited significant antioxidant activity along with moderate SPF potential, making them suitable for sunscreens. Additionally, a topical gel containing menthol, thymol, phloretin and ferulic acid was effective in reducing halitosis in dogs, showcasing its multifunctional potential in oral care [50, 51]. Blackberry and raspberry extracts, formulated into emulsions, displayed high SPF values (54.57 and 37.32, respectively), supporting their role as natural UV protectants. Similarly, a resveratrol–niacinamide–GHK peptide formulation reduced oxidative stress markers and increased NRF2 protein expression, offering protection against environmental skin ageing [45, 90].

Innovations in biopolymer‐based delivery systems have also enhanced antioxidant stability and penetration. *Epilobium angustifolium* extracts infused into bacterial cellulose (BC) membranes demonstrated deep skin penetration, confirming their efficacy as topical carriers. Additionally, *Philadelphus coronarius* extracts incorporated into an ointment exhibited antimicrobial activity, effectively inhibiting *Escherichia coli* and *Staphylococcus aureus* [57, 83].

Targeted treatments for skin conditions have benefited from plant‐based formulations. *Ocimum basilicum* and *Trifolium pratense* extracts in a hydrogel effectively treated psoriasis, while cream and ointment formulations were beneficial for eczema and hypertrophic scars. Furthermore, honey‐based formulations using Western Australian and New Zealand Manuka honey demonstrated strong antioxidant and antibacterial properties, supporting skincare and Wound‐healing applications [31, 85]. A body cream containing coffee silverskin (CS) and *Medicago sativa* extracts confirmed biocompatibility, microbiological safety and commercial viability [32].

Additionally, gallic acid conjugated with E‐polylysine improved oxidative stability in biopolymer‐based emulsions, ensuring prolonged antioxidant efficacy. A periodontal hydrogel containing carvacrol and magnolol significantly reduced oxidative stress markers (MDA, CAT) and inflammation in diabetic‐periodontitis rats [77, 78]. Eucalyptol (5%), formulated into an ointment, reduced edema, improved epidermal integrity and increased antioxidant markers (SOD, catalase), while downregulating inflammatory cytokines (IL‐1β, IL‐6, TNF‐α), confirming its therapeutic Wound‐healing potential. A proprietary antioxidant blend containing green tea polyphenols, Coenzyme Q10, ascorbyl tetraisopalmitate and resveratrol also accelerated skin recovery and reduced inflammation [48, 75].

Finally, *Rhodomyrtus tomentosa* leaf extract, obtained via microwave‐assisted extraction (MAE), exhibited strong antioxidant and anti‐inflammatory properties by inhibiting nitric oxide (NO) production and downregulating IL‐6 and IL‐1β, supporting its role in anti‐inflammatory skincare formulations [91].

Table 4 presents antioxidants with the highest extraction yields, highlighting optimized techniques that enhance bioactive compound retention and bioavailability. Encapsulation‐based extractions, such as quercetin‐loaded mixed micelles (94% efficiency) and Ilex paraguariensis nanoparticles, maximize stability and bioactivity. Fermentation techniques, as seen in Chestnut inner shell and *Magnolia officinalis* bark extracts, increase phenolic and catechin content, improving antioxidant potential. Ultrasound and hydroethanolic extractions (e.g. edible mushrooms, Echinacea and *Medicago lupulina*) yield high concentrations of polyphenols and flavonoids. Macroalgae, cyanobacteria and phlorotannins from *Agarum cribrosum* demonstrate marine‐derived antioxidants' efficiency. The table reflects a trend towards eco‐friendly solvents, advanced ultrasonic methods and fermentation to enhance antioxidant yields for pharmaceutical, cosmetic and nutraceutical applications.

**TABLE 4:** High‐yield antioxidants: Extraction techniques and efficiency.

Antioxidant	Extraction technique & yield	References
Quercetin‐loaded mixed micelles	Polyoxazolines (POx)‐based mixed micelles; encapsulation efficiency: 94 ± 4%, drug loading: 3.6 ± 0.2%	[11]
Grape pomace polyphenols	Natural deep eutectic solvent (NaDES) extraction; BET‐CA solvent showed the best permeation performance	[6]
Chestnut inner shell extract (fermented by *Monascus kaoliang*)	Solid‐state fermentation (SSF) and submerged fermentation (SMF) with *Monascus kaoliang*; SMF yielded higher phenolic acids (395.0 μg/g), SSF yielded more catechins (344.3 μg/g)	[12]
Edible mushroom extracts	Hydroethanolic extraction via ultrasound‐assisted extraction; best extract incorporated into a cosmetic cream	[7]
*Buddleia davidii* extract (Verbascoside & VPP)	Extraction from *Buddleia davidii* meristematic cells; semi‐synthetic derivatization to VPP (acyl derivative) for improved stability	[65]
Mucuna seed EXTRACTS	Ethanolic extraction; M. pruriens had highest L‐DOPA (75.94 mg/100 mg extract); MGG had highest phenolic (56.73 mg GAE/g) and flavonoid (1030.11 mg quercetin/g) content	[24]
Cyanobacteria extracts	Cyanobacteria extracts analysed for total proteins, carotenoids, phenols and phycobiliproteins; different strains demonstrated variable antioxidant properties	[28]
*Magnolia officinalis* bark extract (fermented by *Aspergillus niger*)	*Magnolia officinalis* bark extracted using distilled water, 95% ethanol and methanol; fermented methanol extract had highest tyrosinase inhibition and antioxidant activity	[13]
Red marine macroalgae extracts	Aqueous and hydroethanolic extraction of six red marine macroalgae; water extraction yielded better correlation with bioactivity	[34]
*Arbutus unedo* phenolic extract	Water reflux extraction had the highest yield of phenolics; best antioxidant and anti‐tyrosinase activity	[22]
*Agarum cribrosum* phlorotannins	Ultrasonic extraction of phlorotannins from *Agarum cribrosum*; ethyl acetate (EtOAc) fraction had highest total phlorotannin content (19.59 mg PGE/g)	[27]
*Echinacea purpurea* glycerolic extract	Ultrasound‐assisted glycerol extraction of *Echinacea purpurea*; optimization using Box–Behnken design for efficiency	[18]
*Ilex paraguariensis* (Yerba mate) extract	High‐stirring ethanol extraction of *Ilex paraguariensis* followed by nanoparticle encapsulation; polycaprolactone‐based nanoparticles preferred	[20]
*Malus sylvestris* (Wild apple) fruit extract	Maceration, percolation, Soxhlet and ultrasonic extraction; solvents used: 70% ethanol, 45% and 80% propylene‐glycol, distilled water, sunflower oil, olive oil	[58]
*Medicago lupulina* (Black Medick) extract	Ultrasound‐assisted extraction of *Medicago lupulina* using glycerol and polypropylene glycol; optimized via Box–Behnken design	[64]
Fucus vesiculosus extract	Water (FV‐WE) and 67% ethanol (FV‐EE) extracts; Phlorotannin content: 7–14% dry extract; higher total phenolic content in yellow/brownish algae.	[10]
*Dianella ensifolia* extract	Extraction of *Dianella ensifolia* for DP (1‐(2,4‐dihydrophenyl)‐3‐(2,4‐dimethoxy‐3‐methylphenyl) propane); high DPPH inhibition activity	[61]
*Tinospora cordifolia* nanoparticles	Nanoparticle formulation of *Tinospora cordifolia* extract; average particle size 182.9 ± 3.8 nm for improved dispersion and bioavailability	[81]
*Calendula officinalis* extract	Dry *Calendula officinalis* extract incorporated into hydrophilic cream; optimal extract concentration for antioxidant effect: 0.9%	[55]
*Helichrysum italicum* extract	Hydroxypropyl‐β‐cyclodextrin (HP‐β‐CD)‐assisted extraction of *Helichrysum italicum*; two extracts prepared: OPT‐1 (phenolic acid‐rich), OPT‐2 (phenols & flavonoids‐rich)	[14]

### Emerging green extraction technologies for bioactive recovery

#### Supercritical CO_2_ extraction (SC‐CO_2_)

Supercritical CO_2_ extraction (SC‐CO_2_) employs carbon dioxide above its critical point (31.1°C, 73.8 bar), where it behaves as a supercritical fluid with gas‐like diffusivity and liquid‐like solvating power. This facilitates the selective extraction of non‐polar to moderately polar compounds, while preserving thermolabile constituents. For example, SC‐CO_2_ extraction of oleoresin from *Zingiber officinale* (Peruvian ginger) at 50°C and 250 bar achieved a yield of 25.99 ± 0.13 mg/g dry weight, with high total polyphenol content (171.65 ± 2.12 mg GAE/g extract) and strong antioxidant activity (IC_50_ = 1.02 ± 0.01 mg/mL; FRAP = 368.14 ± 60.95 mg Trolox/g) [92]. Similarly, extraction of *Curcuma longa* at 32°C and 160 bar yielded 5.88 ± 1.09%, with phenolic content of 155.00 ± 0.81 mg GAE/g extract and curcumin concentration of 4.65 mg/L [93]. Optimized SC‐CO_2_ extraction of *Labisia pumila* via response surface methodology (RSM) resulted in high yields of phenolic compounds, including gallic acid (1.2650 ± 0.10% g/g), methyl gallate (0.441 ± 0.29% g/g) and caffeic acid (1.382 ± 0.37% g/g) [94]. SC‐CO_2_ is widely applied for extracting essential oils [95], antioxidants [96] and diverse bioactive compounds from plant materials such as seeds, leaves, fruits and rhizomes [97, 98]. A bibliometric analysis from 1995 to 2024 confirms SC‐CO_2_ as a major research focus in essential oil extraction, particularly in countries like China and Italy [99]. Overall, SC‐CO_2_ is recognized for producing solvent‐free, thermally stable extracts suitable for applications in the food, pharmaceutical and cosmetic industries [100].

#### Subcritical water extraction (SWE)

Subcritical water extraction (SWE) utilizes water at elevated temperatures (100–374°C) under sufficient pressure to maintain its liquid state. Under these conditions, the dielectric constant of water decreases, enhancing its ability to dissolve less polar compounds and eliminating the need for organic solvents. SWE has been effectively applied to extract tannins, flavonoids, volatile oils, anthraquinones and lactones from pharmaceutical plant materials [101]. In a study using amino acid–glucose mixtures (lysine, arginine, alanine) at 200°C and 100 bar for 20 min, SWE facilitated Maillard reactions, yielding neoantioxidants and browning products with strong antioxidant activity, as determined by ABTS and ORACFL assays [102]. Additionally, SWE has shown strong potential for extracting polar and semi‐polar bioactives—including polyphenols, flavonoids and amino acids—from both terrestrial plants and algae, while preserving their biological functionality [103]. Recent reviews have recognized both SC‐CO_2_ and SWE as sustainable, selective and non‐toxic alternatives to traditional organic solvent‐based extraction methods for antioxidants and bioactive compounds [100].

#### Supramolecular solvent extraction (SUPRAS)

Supramolecular solvent extraction (SUPRAS) is based on the spontaneous self‐assembly of amphiphilic molecules into nanostructured liquids, triggered by environmental stimuli such as pH changes, temperature shifts or the presence of electrolytes. These supramolecular solvents support efficient liquid–liquid microextraction across a wide range of analytes, offering enhanced solubilization and compatibility with chromatographic and electrophoretic techniques [104]. For example, SUPRAS formed from Triton X‐114 and the ionic liquid [C14mim]Br enabled the selective extraction of red polyketide pigments from *Talaromyces amestolkiae*, achieving a high partition coefficient (*K* = 14.69 ± 0.15) and over 70% recovery in a single step [105]. In another application, ultrasound‐assisted SUPRAS extraction using octanoic acid‐based reverse micelles in an ethanol–water system facilitated efficient recovery of phenolic acids from *Prunella vulgaris*, surpassing conventional solvents in antioxidant yield. Molecular dynamics simulations confirmed enhanced hydrogen bonding and encapsulation mechanisms [106]. SUPRAS technology has also proven useful for environmental monitoring; a system using SDS and TBABr (1:3 molar ratio) enabled microextraction of phenols from water samples, achieving enrichment factors of 25–64 and recoveries between 82% and 105%, all without heating or organic solvents [107].

#### Cloud point extraction (CPE)

Cloud point extraction (CPE) utilizes non‐ionic surfactants that form coacervate‐rich phases when heated above their cloud point temperature, allowing selective partitioning of target compounds into the surfactant‐rich phase. Lecithin, a natural food‐grade surfactant, has been used for extracting phenolic compounds from wine sludge. Under optimized conditions (5% w/v lecithin, pH 3, 40°C and three recovery stages), the extract demonstrated strong antioxidant activity evaluated via DPPH, Rancimat and DSC assays [108]. In another application, Triton X‐100 was employed to extract phenolics from olive mill wastewater following ultrafiltration. At 90°C and 10% w/w surfactant concentration, the recovery reached 66.5 ± 3.2%, with process efficiency assessed through response surface methodology [109]. Additionally, Triton X‐114 enabled selective extraction of aloe anthraquinones, achieving a 96.93% yield and 70.35% reverse recovery via pH adjustment, all with minimal chemical input [110].

Beyond CPE, other advanced green extraction techniques are gaining momentum across food, pharmaceutical and analytical sectors. Ultrasound‐assisted extraction (UAE) effectively recovered polyphenols and anthocyanins from grape pomace, yielding 115.3 ± 4.7 mg GAE/g TPC and 21.6 ± 1.2 mg C3G/g TAC under optimized conditions [111] Microwave‐assisted extraction (MAE) from onion peels achieved high yields of flavonoids (89.5 ± 2.6 mg QE/g) and phenolics (130.8 ± 3.9 mg GAE/g), while significantly reducing solvent use and extraction time to under 10 min [112]. Pressurized liquid extraction (PLE), also known as accelerated solvent extraction, enabled high‐throughput recovery of saponins from quinoa husks with a yield of 38.2 ± 1.4 mg/g under optimal conditions (100°C, 12 MPa, 70% ethanol) [113]. Pressurized hot water extraction (PHWE), using only subcritical water, presents a green, scalable technique for recovering bioactives and analysing food safety markers from various matrices [114]. Together with SC‐CO_2_, SWE, SUPRAS and CPE, these modern techniques exemplify the ongoing shift towards sustainable, efficient and selective extraction technologies aligned with clean‐label and eco‐conscious trends.

These innovative extraction methods offer sustainable alternatives to conventional solvent‐based techniques, enhancing selectivity, compound stability and environmental performance. Each method—ranging from pressure‐ and temperature‐based systems like SC‐CO_2_ and SWE to surfactant‐ and solvent‐based techniques like SUPRAS and CPE—presents distinct strengths and trade‐offs. Table 5 compares eight advanced extraction technologies in terms of their primary applications, advantages and limitations, highlighting a growing emphasis on eco‐efficiency, scalability and functional ingredient recovery across food, pharmaceutical and analytical domains.

**TABLE 5:** Advanced extraction techniques.

Extraction method	Primary uses/strengths	Advantages	Limitations	References
Supercritical CO_2_ Extraction (SC‐CO_2_)	Efficient extraction of oleoresins, essential oils, curcuminoids, lycopene, antioxidants, lipids; industrially scalable; preserves bioactivity	Solvent‐free, temperature‐controlled, selective, high‐purity extracts; long shelf‐life; green and tunable	High capital cost; less effective for very polar compounds unless co‐solvent used	[92, 93, 94, 95, 96, 97, 98, 99, 100]
Subcritical water extraction (SWE)	Green extraction for polyphenols, flavonoids, amino acids, neoantioxidants; applicable to food, algae, medicinal plants	Eco‐friendly, scalable, efficient; exploits water's tunable polarity; minimal solvent waste	Thermal degradation risk; system requires pressure control and optimization	[100, 101, 102, 103]
Supramolecular solvent extraction (SUPRAS)	Highly selective for pigments, phenols, phenolic acids, organics; analytical‐scale to scalable extraction	Eco‐sustainable; high enrichment; compatible with chromatography; selective recovery	Formulation can be complex; ionic liquids costly; scalability needs validation	[104, 105, 106, 107]
Cloud point extraction (CPE)	Used for phenolic compounds, anthraquinones, antioxidant recovery; one‐step operation; food‐safe surfactants	Low‐cost, surfactant‐based; simple; minimal chemical usage; food/pharma compatibility	Surfactant removal needed for some uses; selectivity depends on conditions	[108, 109, 110]
Pressurized liquid extraction (PLE)	Fast and automated extraction of saponins and bioactives from agri‐by‐products; ethanol‐water solvents	High‐throughput and reproducible; suitable for automation; good recovery from plant residues	Requires high pressure; limited to thermally stable compounds; solvent choice impacts yield	[113]
Microwave‐assisted extraction (MAE)	Rapid extraction of phenolics and flavonoids using microwave heating; low solvent use; short process time	Very fast; reduced solvent and energy use; high yield; minimal degradation	Microwave may degrade heat‐sensitive compounds; uneven heating possible	[112]
Ultrasound‐assisted extraction (UAE)	Enhanced mass transfer for polyphenols and anthocyanins; optimized with ethanol‐water under ultrasound	Improved recovery with less solvent and energy; efficient for high‐value antioxidants from waste	Ultrasound parameters must be optimized; can cause degradation at high intensity	[111]
Pressurized hot water extraction (PHWE)	Green method using water at high pressure and temperature; effective across plant, food and soil matrices	Solvent‐free, scalable; preserves compound integrity; used in sample prep and bioactive extraction	High temperature can degrade sensitive compounds; set‐up requires careful tuning	[114]

## STANDARDIZATION AND EVALUATION OF ANTIAGEING ANTIOXIDANT ASSAYS

The development of natural, plant‐based skincare formulations with antiageing potential requires a rigorous and standardized approach to evaluating antioxidant efficacy, stability, safety and bioavailability. Scientific validation of these products involves comprehensive testing methodologies including phytochemical profiling, in vitro and in vivo antioxidant evaluations, enzyme inhibition studies, formulation and delivery assessments, as well as cytotoxicity and clinical validation. Key antioxidant assays such as DPPH (2,2‐diphenyl‐1‐picrylhydrazyl), FRAP (ferric reducing antioxidant power), ABTS and TRAP are commonly employed to quantify free radical scavenging capacity and reducing power and to benchmark results against reference antioxidants like vitamin C, Trolox and gallic acid. This integrated evaluation framework ensures that botanical extracts meet both scientific standards and consumer expectations for performance and safety.

### Phytochemical profiling and bioactive compound analysis

The foundation for evaluating antioxidant efficacy lies in a thorough analysis of the phytochemical composition of plant extracts. Polyphenols, flavonoids, anthocyanins, tannins and phlorotannins are the key bioactive constituents responsible for antioxidant, antiageing and therapeutic activity. Quantitative and qualitative profiling of these compounds is conducted using advanced analytical methods including high‐performance liquid chromatography (HPLC), gas chromatography–mass spectrometry (GC–MS), nuclear magnetic resonance (NMR) and liquid chromatography–tandem mass spectrometry (LC–MS/MS) [22, 24, 27, 31, 40, 41, 49, 79, 86, 88].

To preserve activity and maximize extraction efficiency, sustainable and optimized methods such as natural deep eutectic solvents (NaDES), ultrasound‐assisted extraction, reflux and microwave‐assisted techniques are employed [6, 22, 27, 40, 91]. The functional stability of antioxidant compounds under environmental stress is further assessed through simulated sunlight exposure studies, singlet oxygen detection and ergosterol oxidation assays, providing insights into oxidative degradation and photostability [86].

### Quantification and characterization of essential antioxidant compounds

Accurate quantification and molecular characterization of bioactive antioxidants is essential for standardization. Advanced analytical techniques, including LC–MS, GC–MS, HPLC and NMR, are used to comprehensively identify and quantify key antioxidant compounds such as phenolics, flavonoids and carotenoids.

#### Identification and quantification of phenolics and flavonoids

Phenolics and flavonoids are major contributors to antioxidant activity in plant, algal and fungal extracts. In *Jasminum sambac* cell extract (JasHEx), phenolic acid derivatives, lignans and triterpenes were identified through mass spectrometry [2]. *Ximenia americana var. caffra* leaf extract was shown to contain 23 secondary metabolites, including flavonol glycosides, flavone glycosides and flavonol diglycosides, characterized via HPLC‐PDA‐ESI‐MS/MS [5]. Edible mushrooms contained p‐hydroxybenzoic acid and gallic acid, with concentrations quantified at 0.010–2.554 μg/g DW and 0.032–0.112 μg/g DW, respectively [7]. In *Mucuna* species seed extracts, HPLC and GC–MS identified L‐DOPA (75.94 mg/100 mg extract), total phenolics (56.73 mg gallic acid/g extract) and flavonoids (1030.11 mg quercetin/g extract) [24]. In *Bergia ammannioides*, HPLC detected β‐sitosterol, lupeol, cyclolaudenol, cycloartenol, quercetin, ellagic acid and kaempferol‐3‐O‐rhamnoside [38].

Polyphenols and flavonoids were also confirmed in *Vitis vinifera* and *Vitis labrusca* grape leaves, with flavonoid fractions showing highest antioxidant activity [21]. *Arbutus unedo* extract exhibited high phenolic and flavonoid content, with hyperoside as the main constituent detected via HPLC‐DAD [22]. *Medicago lupulina* polyphenolic extract revealed apigenin, kaempferol, luteolin, quercetin, caffeic acid, ferulic acid and coumestrol [64]. In *Cecropia pachystachya*, HPLC identified orientin (66.5 μg/mg), isoorientin (118.8 μg/mg) and chlorogenic acid (5.4 μg/mg) [63].

#### Carotenoid analysis and quantification

Carotenoids, crucial for oxidative stress reduction and UV protection, are quantified using HPLC‐UV, GC–MS and spectrophotometry. In *Calendula officinalis*, total carotenoid content in cream formulation was 0.73 ± 0.04 mg/100 g, primarily β‐carotene [55]. Topical and systemic carotenoid delivery resulted in measurable accumulation in human skin and fat tissue, indicating sustained bioavailability [46]. *Cyanobacteria* extracts were rich in zeaxanthin (53.08 μg/mg) and β‐carotene (47.89 μg/mg) as quantified by HPLC [28]. In *Hydnocarpus anthelmintica*, HPLC identified β‐carotene, luteolin and genkwanin, linked to MAPK and p53 regulation [79]. Crocin from saffron demonstrated controlled skin diffusion and was confirmed via HPLC‐UV [115].

#### Structural characterization via LC–MS and NMR

For precise structural elucidation, LC–MS and NMR provide confirmation of antioxidant compound identity. In *Agarum cribrosum*, HPLC‐DAD‐QMS and NMR identified trifuhalol A in phlorotannin‐rich fractions [27]. Western Australian honey was profiled using HPTLC with DPPH and FRAP to confirm polyphenolic content [31]. *Brazilian red propolis* analysis via LC–MS revealed compounds linked to collagen modulation, MMP‐9 inhibition and VEGF expression in wound healing [30]. Additional 1H‐NMR studies confirmed fisetin, retinol, cyanidin, hesperetin, caffeic acid, luteolin, rutin, vanillic acid, ascorbic acid, apigenin, epigallocatechin gallate, rosmarinic acid, myricetin and kaempferol, alongside ergosterol peroxide formation [49]. Other studies identified 6‐methoxyflavonol diglycosides and hydroxy‐polyenoic fatty acids via HPLC–MS/MS and NMR, demonstrating their antioxidant potential [42].

### Standard antioxidant assays: DPPH, FRAP, ABTS and TRAP

Once bioactive compound profiling is complete, antioxidant efficacy is assessed using standardized assays. DPPH and FRAP are widely adopted for measuring radical scavenging and reducing power, often reported as IC50 or relative to vitamin C, Trolox or gallic acid standards. Multiple extracts showed potent antioxidant values: *Ximenia americana var. caffra* (DPPH IC50 = 5 μg/mL; FRAP IC50 = 18.32 μg/mL) [5], *Magnolia officinalis* (DPPH IC50 = 12 μg/mL) [13] and *Bergia ammannioides* (DPPH IC50 = 10.25 μg/mL; ABTS and superoxide active) [38]. *Pandanus amaryllifolius* showed stronger lipid peroxidation inhibition than vitamin C [67], and *Benitaka grape* peel showed DPPH = 92.08% and ABTS = 86.85% [68]. Blueberry nanoberries retained antioxidant activity post‐encapsulation with low cytotoxicity [44], and Brazilian red propolis demonstrated activity in SOD, MPO, GSH and GR assays [30].

### Time‐dependent activity and antioxidant stability

The time‐dependent activity and long‐term antioxidant stability of botanical extracts are essential to determine their lasting efficacy in skincare formulations. For instance, *Malus sylvestris* (wild apple) fruit extract displayed a DPPH scavenging capacity ranging from 8.12% to 78.43% and an FRAP range of 2.13–7.65 mM Fe^2+^ over time, reflecting consistent reducing power [58]. Similarly, *Calendula officinalis* hydro‐ethanolic extract exhibited DPPH‐based antioxidant activity, although its SPF value was 1.75, suggesting limited photoprotective ability [62]. *Hylocereus polyrhizus* (dragon fruit) peel extract demonstrated ABTS scavenging (IC50 = 36.39 μg/mL) and SPF values of 35.02 at 1 mg/mL, confirming its utility as a broad‐spectrum UV protector [23]. *Crataegus monogyna* extract maintained over 90% DPPH and ABTS stability over 90 days, reinforcing its long‐term antioxidant viability [82].

### Enzyme inhibition and photoprotective evaluation

In addition to antioxidant capacity, enzyme inhibition assays are used to evaluate the antiageing effects of extracts. These include assessments against enzymes such as tyrosinase (involved in melanin synthesis), collagenase, elastase, hyaluronidase and matrix metalloproteinases (MMPs), which are implicated in skin degradation, wrinkle formation and loss of elasticity [13, 17, 24, 27, 29, 79, 88]. Cyanobacteria extracts exhibited superoxide radical scavenging (IC50 = 63.24 μg/mL), elastase inhibition (IC50 = 380.50 μg/mL) and hyaluronidase inhibition, demonstrating multifunctional bioactivity [28]. *Bunium alpinum* extract, rich in flavonoids and phenolics, exhibited DPPH radical scavenging activity of 20.65 mg VCE/g and total antioxidant activity of 196.69 mg VCE/g. It also showed SPF values of UVB = 14.70 and UVA = 32.80, indicating additional photoprotective benefits [41]. *Pimenta pseudocaryophyllus* showed high radical scavenging activity (DPPH IC50 = 4.75 μg/mL, ABTS IC50 = 3.0 μg/mL), and its FRAP activity was equivalent to 0.046 μmol/L Trolox [84].

### Formulation stability, bioavailability and skin delivery

Formulation stability, delivery and bioavailability studies are crucial for translating antioxidant efficacy into viable skincare products. Nanoformulations—such as nanoemulsions, liposomes, niosomes, ethosomes and nanostructured lipid carriers—are used to enhance skin penetration and protect labile compounds [9, 11, 15, 20, 25, 43, 44, 52, 53, 54, 69, 71, 72, 73, 80, 89, 115]. Stability testing evaluates pH, viscosity, colour, spreadability and storage resilience under various environmental conditions [29, 31, 40, 43, 52, 60]. Penetration and release kinetics are assessed using Franz diffusion cells, tape‐stripping techniques and in vitro skin models [6, 9, 15, 20, 25, 38, 40, 47, 52, 71, 73]. Encapsulation enhances bioavailability and longevity of active compounds like caffeic acid, ferulic acid, retinol, flavonoids, polyphenols and gallic acid, which are otherwise prone to degradation [44, 69, 78]. Emulsification and retinol stability studies further ensure the product retains its activity throughout its shelf‐life [78].

### Cytotoxicity, biocompatibility and wound healing

Ensuring safety and skin compatibility is paramount. Cytotoxicity and biocompatibility are assessed via MTT assays on a range of human and animal cell lines, including fibroblasts (CCD‐966SK, L929, NIH3T3), keratinocytes (HaCaT), melanocytes (HEMn), macrophages (RAW 264.7) and melanoma cells (A375) [11, 13, 29, 44, 54, 89, 91, 115]. Anti‐inflammatory effects are measured through cytokine inhibition (IL‐6, IL‐1β, TNF‐α, CXCL10), along with COX‐2, iNOS suppression and NRF2 activation [29, 30, 42, 90, 91]. In vivo animal models—such as carrageenan‐induced paw edema, CFA‐induced hyperalgesia and formalin pain tests—are employed to assess anti‐inflammatory effects [15, 39, 42, 63]. Wound‐healing activity is evaluated in Wistar rats, using histological and immunohistochemical analysis to monitor collagen I/III synthesis, VEGF expression and MMP‐9 modulation [30, 38].

### Clinical trials and consumer evaluation

To confirm in‐use efficacy, human clinical studies and consumer evaluations assess parameters such as skin hydration, elasticity, pigmentation, radiance and fine‐line reduction [46, 76]. Resonance Raman spectroscopy enables non‐invasive measurement of antioxidant accumulation in the skin [46]. RNA sequencing and inflammatory gene expression profiling help validate the antioxidant effect against pollution‐induced oxidative damage [90]. Consumer studies, including UVB‐induced tan fading trials, provide real‐world comparisons between plant‐based formulations and conventional products [61].

### Periodontal and Oral health applications

Beyond dermatological applications, plant‐based antioxidants also demonstrate potential in oral health. Their efficacy in reducing oxidative stress and inflammation in periodontal diseases is confirmed through studies measuring malondialdehyde (MDA), catalase (CAT) activity and IL‐1β levels in periodontitis models [77]. Clinical evaluations also explore benefits for halitosis reduction, gum health improvement and plaque prevention.

### Comparative benchmarking with reference antioxidants

Antioxidant capacity is routinely benchmarked against reference compounds to ensure potency and standardization. Lacto‐fermented, microencapsulated guava juice powder demonstrated a DPPH IC50 of 0.015 mg/mL, comparable to ascorbic acid (IC50 = 0.018 mg/mL) [60]. Similar comparative studies using Trolox and gallic acid equivalents confirm the relative efficacy of a wide range of botanical extracts [28, 41, 84], providing a standardized scale for evaluating novel plant‐based antioxidants in cosmetic and therapeutic formulations.

Table 6 outlines the diverse methods used to evaluate the efficacy, stability and safety of leaf‐derived antioxidants in skincare formulations. Stability testing confirms the long‐term retention of antioxidant activity, pH consistency and microbiological safety. Antioxidant activity assays such as DPPH, ABTS and FRAP validate the free radical scavenging potential, while enzyme inhibition studies highlight anti‐ageing benefits by preventing collagen and elastin degradation. Photoprotective and cytotoxicity tests ensure UV protection and skin compatibility. Advanced bioactive compound profiling and nanoformulation characterization enhance product standardization and effectiveness. Clinical trials and consumer feedback confirm real‐world efficacy, supporting hydration, wound healing and oxidative stress reduction. Overall, these testing methods ensure antioxidant formulations are effective, safe and optimized for skincare applications.

**TABLE 6:** Testing methods for antioxidant efficacy and safety.

Testing and evaluation patterns	Importance of the test	Key tests, findings and technical details	References
Stability testing	Ensures long‐term effectiveness of antioxidant formulations, preventing degradation and maintaining efficacy	Stability maintained over 6–12 months; antioxidant activity retention >85%; pH variation <0.2 units; viscosity stability ±5%; microbiological quality confirmed	[1, 8, 31, 32, 36, 55, 62]
Antioxidant activity	Confirms the ability of antioxidants to neutralize free radicals, reducing oxidative stress and skin damage	DPPH IC50 values: 0.15 μg/mL (grape pomace), 36.39 μg/mL (dragon fruit), 80 μg/mL (Heliotropium extract). ABTS and FRAP assays confirmed 90% + radical scavenging in multiple extracts	[3, 6, 18, 19, 23, 37, 75]
Enzyme inhibition (antiageing)	Demonstrates the anti‐ageing potential by preventing collagen breakdown and reducing wrinkles and hyperpigmentation	Collagenase inhibition: 78.5% (Stevia extract); elastase inhibition: 69% (*Echinacea purpurea*); tyrosinase IC50: 30 μg/mL (Magnolia bark)	[13, 17, 18, 64, 66, 79]
Cytotoxicity and biocompatibility	Ensures that antioxidant formulations do not harm skin cells and are safe for prolonged topical use	Cytotoxicity <20% (blueberry polyphenols on HaCaT cells); fibroblast viability maintained at >95% with quercetin‐loaded liposomes; safe for human topical use	[13, 44, 57, 81, 91]
Photoprotective testing	Confirms the ability of antioxidants to prevent UV‐induced skin damage, reducing risk of photoageing and sunburn	SPF values: 54.57 (blackberry extract), 35.02 (dragon fruit), 18.56 (grape peel extract). UVA/UVB absorbance efficiency 80–90% for multiple plant‐based extracts	[21, 23, 41, 45, 68, 72]
Wound healing and anti‐inflammatory tests	Demonstrates effectiveness in promoting skin regeneration and reducing inflammation in dermatological applications	Wound contraction improved by 85% (*Bergia ammannioides* extract); cytokine reductions (IL‐6, TNF‐α); increased collagen deposition by 40%	[26, 30, 38, 48, 56]
In vivo skin hydration and erythema	Confirms ability to strengthen the skin barrier, increase hydration and reduce redness and irritation	TEWL (transepidermal water loss) reduced by 30% in SDS‐damaged skin; 90% user satisfaction with hydration levels; erythema reduced by 18.5%	[1, 3, 32, 71, 75]
Bioactive compound analysis	Determines the chemical profile of antioxidants, helping to standardize and optimize extraction and formulations	Phenolic and flavonoid profiling via HPLC and LC‐MS/MS; phenolic content increased by 40% after fermentation. Identified active compounds responsible for antioxidant activity	[5, 12, 13, 14, 22, 37, 58]
Nanoformulation characterization	Ensures improved skin penetration, prolonged antioxidant release and enhanced formulation effectiveness	Vesicle size: ~200 nm (average for optimized nanoemulsions); encapsulation efficiency >85%; sustained release of antioxidants for 24–84 h	[11, 15, 52, 71, 74]
Skin penetration studies	Validates bioavailability of antioxidants in the skin, ensuring better therapeutic effectiveness	Franz diffusion cells, ex vivo permeation and tape‐stripping techniques confirmed deeper epidermal and dermal antioxidant delivery	[9, 20, 25, 57, 73, 115]
Clinical trials and consumer feedback	Ensures formulations deliver noticeable benefits to users, improving efficacy, marketability and acceptance	Clinical studies confirmed hydration improvement, reduction in fine lines and 90% consumer satisfaction with skincare formulations	[32, 76]
Oxidative stress and ROS reduction	Confirms ability to counteract oxidative stress, preventing premature ageing and skin damage	SOD, CAT and GSH levels increased; ROS levels reduced by 40–60% in fibroblast cells treated with antioxidants	[19, 30, 38, 48, 75, 77]
Advanced imaging and spectroscopy	Provides molecular insights into formulation stability, ensuring better consistency and efficacy	Cryo‐SEM, TEM, FTIR and DSC used for structural and physicochemical analysis of antioxidant formulations	[1, 53, 89, 115]

## VALIDATION OF NATURAL‐BASED ANTIOXIDANTS

### In‐vitro studies on natural‐based antioxidants

In‐vitro studies have demonstrated the potent antioxidant, anti‐ageing and photoprotective properties of natural antioxidants, reinforcing their potential for cosmeceutical and therapeutic applications. These studies evaluate bioavailability, stability, enzyme inhibition and delivery systems, optimizing formulations for skincare and health‐related uses.

#### Antioxidant & Anti‐Aging Properties

*Moringa oleifera* hydroalcoholic fractions improved radical scavenging, skin hydration and lipid content when formulated into nanoparticles. Similarly, *Ajuga spectabilis* extracts exhibited strong free radical scavenging, anti‐inflammatory activity and tyrosinase inhibition, supporting their role in wrinkle prevention and skin whitening [3, 4]. While *Fucus vesiculosus* extracts provided UV protection, their low SPF values limited their potential as stand‐alone sunscreens. Conversely, *Helichrysum italicum* extracts inhibited skin‐ageing enzymes (collagenase, elastase), reinforcing their potential in anti‐ageing skincare [10, 14]. *Calendula officinalis* cream effectively scavenged hydrogen peroxide radicals due to its high carotenoid and polyphenol content, ensuring formulation stability. Marine macroalgae, including *Phycocalidia acanthophora* and *Porphyra linearis*, demonstrated collagenase inhibition and photoprotective effects, further validating their role in skincare applications [34, 55]. Additionally, *Echinacea purpurea* ultrasound‐assisted extracts enhanced keratinocyte wound healing and enzyme inhibition, while *Sargassum thunbergii* ethanolic extract significantly reduced ROS levels and inhibited collagenase and elastase, reinforcing its potential in skincare formulations [18, 29]. *Agarum cribrosum* phlorotannins were also found to inhibit collagenase, elastase and tyrosinase, with Trifuhalol A identified as the key active compound [27].

#### Bioavailability & Delivery Systems

Grape pomace polyphenols extracted using natural deep eutectic solvents (NaDES) demonstrated enhanced skin permeation in 3D human keratinocytes, improving bioavailability. Additionally, polyoxazoline‐based lipid nanocapsules (LNC‐POx) achieved a threefold increase in quercetin loading compared to PEG‐LNC, thereby enhancing antioxidant activity under oxidative stress conditions [6, 54]. Quercetin‐loaded magnetoliposomes (QMLs) developed with iron oxide nanoparticles improved solubility and antioxidant properties, whereas rutin‐loaded ethosomes achieved high encapsulation efficiency (80.2%) but exhibited limited penetration in ex vivo studies [9, 53]. Curcumin‐loaded niosomes displayed potential for oral cancer prevention by inhibiting KB oral cancer cell growth, while layer‐by‐layer emulsion coatings improved retinol stability, preserving its antioxidant activity [70, 78]. Additionally, epigallocatechin‐3‐gallate (EGCG) stabilized in topical creams with co‐antioxidants demonstrated enhanced photostability and sustained antioxidant efficacy [86].

#### Cosmeceutical & Antimicrobial Applications

Hydroethanolic extracts from mushrooms (*Agaricus bisporus*, *Boletus edulis*, *Lentinula edodes*, *Pleurotus ostreatus*) exhibited antibacterial, antioxidant and hyaluronidase inhibition properties, confirming their potential in cosmetic applications. Fermented *Magnolia officinalis* bark extracts, produced using *Aspergillus niger*, increased phenolic content, radical scavenging activity and antibacterial efficacy against MRSA [7, 13]. *Malus sylvestris* (wild apple) extracts contained high polyphenol content and antioxidant activity, supporting their use in both cosmetics and the food industry. *Mucuna* species seed extracts also exhibited antimicrobial, antioxidant and anti‐ageing properties, with *Mucuna pruriens* displaying the highest L‐DOPA content [24, 58]. Additionally, *Stevia rebaudiana* demonstrated the highest phenolic content, while *Morus alba* exhibited significant anti‐inflammatory activity among 16 herbal extracts screened for cosmeceutical applications [16].

#### Photoprotective & Sunscreen Potential

Several natural extracts have demonstrated strong photoprotective properties. Blackberry and raspberry extracts exhibited high SPF values (54.57 and 37.32, respectively), confirming their potential for natural sunscreen formulations. *Vitis vinifera* (Benitaka grape peel) flavonoid‐rich extract, incorporated into a stable sunscreen formulation, provided an in vitro SPF of 18.56 [45, 68]. *Hylocereus polyrhizus* (dragon fruit) peel extract exhibited strong UV absorption, achieving an SPF of 35.02 at 1 mg/mL. Similarly, *Bunium alpinum* butanolic extract displayed UVB and UVA photoprotective activity, with SPF values of 14.70 and 32.80, confirming its broad‐spectrum sun protection potential. Propolis also demonstrated lipid peroxidation inhibition and broad‐spectrum photoprotection, reinforcing its effectiveness in sunscreen formulations [23, 36, 41].

#### Stability & Nanoformulation Optimization

Nanoemulsions containing basil, lemon balm and oregano essential oils, developed via the phase inversion composition (PIC) method, exhibited varying antioxidant activity. Among them, oregano‐based nanoemulsions demonstrated the highest stability over 6 months. Similarly, nanoemulsion formulations incorporating *Arbutus unedo* extracts exhibited optimal pH stability while maintaining strong antioxidant and anti‐tyrosinase activity [8, 22]. A non‐aqueous gel containing curcumin‐loaded nanoparticles (CA‐NP) displayed strong antioxidant activity (IC50 = 5.39 μg/mL, DPPH assay) with enhanced stability, further supporting its use in skincare [74].

#### Safety assessments in formulations

A study analysing 14 common antioxidants identified potential concerns regarding photosensitization. Fisetin, retinol, cyanidin and hesperetin were found to generate singlet oxygen, increasing the risk of UV sensitivity. In contrast, caffeic acid, luteolin, rutin and vanillic acid did not exhibit this effect, making them safer choices for cosmetic formulations [49].

### Ex vivo studies on natural‐based antioxidants

Ex vivo studies provide essential insights into the penetration, bioavailability, stability and efficacy of natural antioxidants in skin models. These studies bridge the gap between in vitro experiments and clinical applications, validating the effectiveness of formulations in cosmeceutical and therapeutic products.

#### Antioxidant, Anti‐Glycation & Anti‐Inflammatory Effects

*Jasminum sambac* cell extract (JasHEx) demonstrated strong anti‐ageing properties by reducing reactive oxygen species (ROS) and inhibiting advanced glycation end‐product (AGE) formation, leading to increased collagen type I production. Meanwhile, rutin‐loaded ethosomes achieved high encapsulation efficiency (80.2%), but ex vivo studies did not confirm significant penetration, highlighting the need for further formulation optimization [2, 9].

#### Enhanced Antioxidant Stability & Retention

Encapsulation techniques have significantly improved the stability and retention of antioxidants. *Ilex paraguariensis* extract, when incorporated into polymeric nanoparticles, enhanced chlorogenic acid stability and ensured prolonged antioxidant activity in the skin for up to 12 h. Similarly, polyphenol‐rich extracts from *Vitis vinifera* and *Vitis labrusca* exhibited selective flavonoid retention in porcine skin, confirming their potential for long‐lasting antioxidant benefits in topical applications [20, 21]. Lipid vesicular gels (LVGs) containing caffeic and ferulic acids demonstrated controlled release, with ferulic acid achieving deeper skin penetration for extended protection. Additionally, quercetin‐loaded chitosomes (liposome‐chitosan hybrids) enhanced antioxidant performance, with chitosan ensuring controlled release and improved stability [52, 69].

#### Skin Penetration & Stability

Delivery systems have also played a crucial role in improving skin penetration and stability. Berberine‐loaded oleosome gel exhibited enhanced skin retention and sustained release in ex vivo models, while in vivo studies further confirmed its antioxidant and anti‐inflammatory effects, supporting its potential for vitiligo treatment. Similarly, *Terminalia arjuna* extract, formulated into emulsions, demonstrated strong antioxidant and anti‐tyrosinase activity with effective skin permeation, highlighting its role in pigmentation control [26, 43]. *Vaccinium myrtillus* (blueberry) polyphenol‐rich extracts encapsulated in ultradeformable liposomes retained 85% of their antioxidant activity, improving bioavailability in ex vivo skin models. Additionally, resveratrol‐loaded ultradeformable vesicular cream significantly increased skin retention (~335.2 μg/cm^2^), further enhancing its antioxidant efficacy [44, 71].

#### Advanced antioxidant delivery systems

Hydrogel‐based antioxidant formulations have shown promising results in controlled release applications. *Baccharis incarum* ethanol extracts incorporated into hydrogels exhibited strong antibacterial and antioxidant properties, with Franz diffusion studies confirming the controlled release of chlorogenic acid and flavonoids, thereby enhancing formulation stability. Similarly, nanostructured lipid carriers (NLCs) for sesamol achieved high encapsulation efficiency (>90%) and prolonged antioxidant activity, making them a promising system for long‐lasting topical applications [40, 80]. The gliadin–sericin complex nanoparticle (G‐LSS) further enhanced phloretin bioavailability, offering UV degradation protection and improved skin penetration, reinforcing its use in photoprotective formulations [89].

#### Enhanced Antioxidant Stability & Delivery

Innovative nanotechnology‐based approaches have also improved antioxidant stability and delivery. Nanostructured lipid dispersions for crocin delivery significantly enhanced antioxidant stability while ensuring controlled skin diffusion, making them ideal for dermatological applications. Additionally, a topical ointment containing *Philadelphus coronarius* extracts exhibited strong antimicrobial and antioxidant properties, confirming its dermatological potential [83, 115]. Furthermore, honey‐based formulations using Western Australian and Manuka honey retained high antioxidant and antibacterial activity, supporting their use in Wound‐healing and skincare applications [31].

### In vivo animal studies on natural‐based antioxidants

In vivo animal studies provide critical insights into the efficacy, bioavailability and safety of natural antioxidants in biological environments. These studies evaluate their effects on oxidative stress, inflammation, wound healing, oral health and dermatological applications, reinforcing their potential for use in skincare and therapeutic formulations.

#### Antioxidant & Anti‐Aging Potential

*Ximenia americana var. caffra* leaf extract, rich in secondary metabolites, significantly reduced ROS levels and activated the DAF‐16 transcription factor in *Caenorhabditis elegans*, enhancing oxidative stress resistance and longevity. These findings confirm its dermatological and anti‐ageing potential [5].

#### Anti‐Inflammatory & Wound‐healing Effects

Several natural extracts have demonstrated strong Wound‐healing and anti‐inflammatory properties. *Annona muricata* leaf aqueous extract (AEAM) suppressed ROS release in fibroblasts and reduced inflammation in a TPA‐induced ear inflammation model in mice, reinforcing its potential for inflammation control. Similarly, *Bergia ammannioides* ethanolic extract promoted wound healing in an excision wound model by enhancing collagen content while exhibiting potent antioxidant and anti‐inflammatory properties [38, 116]. In dermatological applications, *Calendula officinalis* cream formulations (10% and 30%) significantly reduced pain and inflammation in rats via opioid receptor pathways, confirming their effectiveness in skin soothing and pain relief [56].

#### Oral Health & Cancer Prevention

A topical gel containing menthol, thymol, phloretin and ferulic acid significantly reduced halitosis in a blinded crossover trial in dogs, indicating strong oral hygiene benefits. Additionally, curcumin‐loaded niosomes demonstrated potent antioxidant and anticancer properties in a carcinogen‐induced rat model, supporting their potential in oral cancer prevention [51, 70].

#### Inflammation & Pain Management

Several plant‐based formulations have demonstrated anti‐inflammatory and pain‐relief properties. *Leonurus sibiricus* ethanol extract (EELs) exhibited strong antioxidant activity, reduced lipid peroxidation and inhibited inflammation markers in a formalin‐induced pain model, confirming its analgesic and anti‐inflammatory effects. Similarly, *Cecropia pachystachya* methanolic extract (CPM) significantly reduced vasodilation, oedema and cell infiltration in a mouse ear inflammation model, reinforcing its therapeutic potential for inflammatory skin conditions [39, 63]. Additionally, *Oxybaphus nyctagineus* extracts effectively reduced hyperalgesia and oedema in rat models, supporting their role in pain relief formulations [42].

#### Bioavailability & Biocompatibility

*Tinospora cordifolia* nanoparticles (~182.9 nm) exhibited enhanced dispersion and absorption of active compounds with no observed cytotoxicity or dermal irritation in a rabbit model, confirming their safety for anti‐ageing skincare applications. Additionally, a niosome‐based gel (AN‐NS2G4) formulated with apigenin significantly improved transdermal delivery and exhibited potent anti‐inflammatory effects in a carrageenan‐induced paw oedema model, supporting its potential for topical inflammation treatment [15, 81].

#### Wound Healing & Skin Repair

Multiple studies confirm the Wound‐healing potential of natural antioxidants. *Heliotropium bacciferum* extract accelerated wound contraction, increased glutathione levels and enhanced antioxidant enzyme activity in Wistar rats, highlighting its role in tissue regeneration. Meanwhile, Brazilian red propolis (BRP) promoted collagen synthesis and reduced inflammation in a rat skin excision model, validating its efficacy as a natural Wound‐healing agent [19, 30]. Additionally, a 5% eucalyptol ointment significantly reduced oedema and inflammation while increasing capillary formation in a third‐degree burn rat model, reinforcing its potential in burn treatment. A periodontal hydrogel containing carvacrol and magnolol significantly reduced oxidative stress markers (MDA) and increased catalase activity in diabetic Wistar rats, confirming its effectiveness in promoting periodontal health [48, 77].

#### UV Protection & Skincare Benefits

Natural compounds have also shown promise in photoprotection and skincare. *Pimenta pseudocaryophyllus* extract exhibited strong antioxidant and UVB‐protective effects, reducing oxidative stress in irradiated mice, supporting its potential as a natural sunscreen ingredient. Furthermore, *Arthrospira platensis* extracts demonstrated antifungal, antioxidant and cytotoxic properties, effectively treating fungal infections when formulated into a topical cream in vivo, confirming their potential for antifungal skincare applications [35, 84].

### In vivo human studies on natural‐based antioxidants

In vivo human studies are essential for validating the efficacy, safety and stability of natural antioxidants in real‐world applications. These studies assess antioxidant accumulation, skin hydration, barrier protection, skin‐lightening effects and treatments for various skin conditions, ensuring their effectiveness in dermatology and cosmeceutical formulations.

#### Antioxidant Stability & Skin Hydration

A *Castanea sativa* leaf extract, formulated into a surfactant‐free base, maintained stability for 6 months while ensuring microbiological integrity. It provided significant moisturization for at least 4 h and was well tolerated, reinforcing its potential for oxidative stress‐related skin conditions. Additionally, a controlled human study using Raman spectroscopy demonstrated that combined topical and systemic antioxidant treatments led to the highest skin antioxidant accumulation. In contrast, systemic treatments alone provided prolonged protection for up to 5 weeks due to gradual release from fat storage [1, 46].

#### Skin‐lightening & UV protection

*Dianella ensifolia* extract (DP) exhibited strong free radical inhibition, reduced lipid oxidation and significantly accelerated UVB‐induced tan fading. It outperformed hydroquinone‐based treatments, confirming its potential as a safer alternative for managing hyperpigmentation and UV protection [61].

#### Skin Recovery & Barrier Protection

A clinical study on Japanese volunteers evaluated a formulation containing ascorbyl tetraisopalmitate, Coenzyme Q_10_, green tea polyphenols and resveratrol. Among these, green tea polyphenols provided the most significant improvement in skin barrier function and inflammation reduction. In a separate 12‐week trial, a night‐time antioxidant blend containing 1% resveratrol, 0.5% baicalin and 1% vitamin E led to improvements in fine lines, wrinkles, firmness, elasticity and dermal thickness (+18.9%), confirming its anti‐ageing benefits [75, 76].

#### Topical treatment for skin conditions

Various botanical formulations have shown condition‐specific skin benefits. Creams, ointments and hydrogels containing *Ocimum basilicum* (basil) and *Trifolium pratense* (red clover) extracts demonstrated efficacy in treating different dermatological conditions. The cream was particularly effective for dyshidrotic eczema, the ointment reduced hypertrophic scars and the hydrogel significantly diminished psoriatic plaques within 6 days. Additionally, a body cream infused with coffee silverskin and *Medicago sativa* (alfalfa) extracts significantly improved skin hydration and was well tolerated, with 90% of participants reporting a positive experience [32, 85].

#### Anti‐Inflammatory & Antimicrobial Benefits

*Rhodomyrtus tomentosa* extract, obtained via microwave‐assisted extraction (RT‐MAE), exhibited strong anti‐inflammatory, antibacterial and antioxidant properties. When assessed in a topical formulation, it demonstrated good biocompatibility in human volunteers, confirming its potential as a safe and effective anti‐inflammatory skincare ingredient [91].

## NATURAL‐BASED ANTIOXIDANTS VERSUS COMMERCIAL FORMULATIONS

Antioxidants play a crucial role in skincare, pharmaceuticals and nutraceuticals, with many natural and bioengineered compounds outperforming or matching commercial formulations in bioavailability, stability and therapeutic effectiveness. Encapsulation technologies such as niosomes, liposomes, nanoemulsions and polymeric micelles have enhanced skin penetration, antioxidant activity and controlled release, making them more effective than free compounds. Plant‐based extracts, including Moringa, Propolis and Mushroom‐derived antioxidants, exhibit stronger antioxidants, anti‐inflammatory and UV‐protective properties than conventional skincare ingredients. Multicomponent formulations, such as resveratrol blends and EGCG stabilizers, offer improved stability and bioactivity, positioning them as promising alternatives in the cosmetic and medical industries. This trend highlights the growing shift towards natural, bioactive and nano‐enhanced antioxidant solutions as viable competitors to traditional market products (Table 7).

**TABLE 7:** Comparative analysis of antioxidants: performance versus commercial formulations.

Antioxidant	Comparison with commercial products	References
*Jasminum sambac* cell extract	Demonstrated superior antioxidant activity and collagen production stimulation	[2]
*Moringa oleifera* leaf extract	Enhanced moisturizing and antioxidant benefits compared to generic formulations	[3]
*Ximenia americana var. caffra* leaf extract	Comparable activity to reference drugs in enzyme inhibition; effective antioxidant and skin‐protecting properties	[5]
Grape pomace polyphenols	BET‐CA outperformed other NaDES formulations in permeation and antioxidant activity	[6]
Edible mushroom extracts	Cream with A. bisporus extract had significantly higher phenolic content and antioxidant activity than the control cream	[7]
Essential oils (Basil, Lemon Balm, Oregano)	OR‐NE had superior stability and antioxidant retention compared to non‐nanoemulsified essential oils	[8]
*Dianella ensifolia* extract	DP‐containing cosmetic formula increased skin discoloration fading rate better than 4% hydroquinone formulations	[61]
Caffeic & ferulic acid‐loaded lipid vesicular gels	Encapsulation of antioxidants improved topical delivery compared to free compounds	[69]
*Calendula officinalis* cream	Ca cream (10% or 30%) reduced inflammation, oedema and pain in multiple inflammation models	[56]
Quercetin‐loaded liposomes & chitosomes	Chitosomes showed enhanced skin retention and antioxidant protection compared to free quercetin	[52]
Quercetin‐loaded magnetoliposomes (QMLs)	QMLs outperformed standard quercetin formulations in antioxidant activity and stability	[53]
Quercetin‐loaded polyoxazoline lipid nanocapsules (LNC POx)	POx‐based LNCs provided three times higher drug loading and better stability than PEG‐based LNCs	[54]
Berberine‐loaded gel‐core oleosomes	BER‐oleosomes had superior topical bioavailability and therapeutic effects compared to standard berberine gel	[26]
Curcumin‐loaded niosomes	Injectable form showed stronger anticancer and antioxidant effects compared to curcumin mouthwash	[70]
Resveratrol‐loaded ultradeformable vesicular cream	Vesicular cream had superior penetration and bioavailability compared to conventional cream	[71]
Trans‐resveratrol microemulsions (RO‐ME, JO‐ME, LO‐ME)	Microemulsions improved trans‐resveratrol delivery compared to aqueous solutions	[72]
Resveratrol nanoemulsions with terpenes	Nanoemulsions showed superior skin penetration over saturated RSV solutions	[73]
Resveratrol‐loaded liposomes & niosomes (Plurol Oleique, Peceol)	Niosomes (Plurol Oleique, Peceol) outperformed liposomes for skin accumulation	[25]
Green tea polyphenols (GTP), ascorbyl tetraisopalmitate, Coenzyme Q_10_, resveratrol	Antioxidant‐based formulations significantly improved skin recovery compared to untreated skin	[75]
*Hylocereus polyrhizus* (red dragon fruit) peel extract	Stronger photoprotective effect compared to conventional UVB protection ingredients	[23]
Propolis extract	Proposed as an active ingredient in commercial sunscreen formulations	[36]
Epigallocatechin‐3‐gallate (EGCG) with co‐antioxidants	Comparison of different stabilizers; α‐lipoic acid significantly reduced degradation compared to vitamin C and butylated hydroxytoluene	[86]
Brazilian red propolis	Compared to collagenase; demonstrated similar or superior effects on wound healing	[30]
Eucalyptol (topical ointment)	Compared with silver sulfadiazine (SS) ointment; similar healing effects observed	[48]
Sesamol (*Sesamum indicum* extract)	Evaluated against reference formulations; NLC‐PLUS demonstrated better bioavailability and controlled diffusion	[80]
Honey‐loaded topical formulations	WA Manuka honey formulations showed higher antioxidant activity than NZ Manuka honey	[31]

## CURRENT LIMITATIONS OF NATURAL‐BASED ANTIOXIDANTS IN SKINCARE

Despite their strong potential in anti‐ageing, photoprotection, anti‐inflammatory effects and skin rejuvenation, antioxidant‐based skincare formulations face several challenges that hinder their clinical application and commercial scalability. Key limitations include photostability, bioavailability, standardization, clinical validation, and cost‐effectiveness, all of which must be addressed for their widespread adoption.

### Photostability and environmental susceptibility

Many botanical antioxidants degrade under UV exposure, heat and oxidation, leading to reduced efficacy over time. This instability poses challenges for their incorporation into cosmetic formulations and sunscreens.

#### Degradation under UV and heat exposure

Studies have shown that *blackberry* and *raspberry* extracts significantly lose their antioxidant and photoprotective effects when exposed to light and heat, limiting their shelf‐life in cosmetic formulations [45]. Similarly, *Calendula officinalis*‐based formulations exhibited low SPF values (~1.75), making them unsuitable as stand‐alone sunscreens without additional UV filters [62]. Even promising natural sunscreens require stabilization to maintain efficacy. For example, *Vitis vinifera* (grape peel) extract formulations experienced gradual SPF degradation during long‐term storage, highlighting the need for improved stabilizers [68]. Essential oils such as geranium, calendula and oregano also provided minimal SPF protection, proving insufficient as primary UV‐blocking agents [50].

#### Variability in marine and botanical antioxidants

Marine macroalgae‐based extracts, such as *Fucus vesiculosus* and red algae (*Phycocalidia acanthophora*), have demonstrated UV‐absorbing properties. However, their phenolic content and photoprotective efficacy vary depending on species and extraction methods, making standardization difficult [10, 34]. Similarly, grape leaf extracts (*Vitis vinifera*, *Vitis labrusca*) showed poor flavonoid penetration into deeper skin layers, reducing their effectiveness as photoprotective agents [49].

#### Pro‐oxidant risks and photodegradation

Some antioxidants may paradoxically become pro‐oxidants under UV exposure, leading to increased oxidative stress rather than protection. For instance, fisetin, retinol, cyanidin and hesperetin have been shown to generate singlet oxygen under UV exposure, potentially contributing to skin damage instead of preventing it [86, 89]. Additionally, polyphenols such as EGCG and phloretin are highly susceptible to photodegradation, requiring stabilization with co‐antioxidants like alpha‐lipoic acid or encapsulation in advanced carriers such as gliadin‐sericin colloidal particles for enhanced stability [21].

#### Challenges in formulation stability

Ensuring formulation stability remains a key challenge for many botanical antioxidants. *Bunium alpinum* extract, despite its high SPF values, lacks long‐term photostability data, making UV‐exposure testing essential for commercial viability [41]. Similarly, *Sargassum thunbergii* ethanolic extract, despite its promising anti‐ageing potential, lacks stability validation, raising concerns about its effectiveness in long‐term applications [29]. Moreover, propolis‐based SPF formulations have shown high batch‐to‐batch variability in UVA/UVB ratios, requiring strict quality control measures to ensure consistency in efficacy [36].

### Lack of large‐scale clinical trials and regulatory approval

Despite the promising potential of antioxidant‐based skincare formulations, most lack large‐scale, randomized, double‐blind human trials, which are essential for obtaining FDA and EMA regulatory approval.

#### Need for clinical validation

While preclinical studies have demonstrated the strong antioxidant and anti‐ageing properties of green tea polyphenols, resveratrol and cyanobacteria extracts, their real‐world dermatological benefits remain unconfirmed due to the absence of placebo‐controlled human trials [28, 35, 75].

#### Reliance on in vitro and animal models

Many existing studies rely on in vitro assays and animal models, making it difficult to establish long‐term safety and efficacy in humans. For example, dual‐application strategies involving both systemic and topical antioxidants, such as carotenoids, require extensive research to determine the optimal dosage, safety profile and effectiveness in large human populations [46].

#### Pending clinical trials for cosmeceutical adoption

Several antioxidant‐rich extracts, including grape pomace extracts, blueberry polyphenol nanocarriers and lacto‐fermented guava juice powder (FGJP), have shown promising results in preliminary studies. However, they require further clinical validation before being widely adopted in cosmeceutical formulations [6, 44, 60].

### Bioavailability and skin penetration challenges

Many plant‐derived antioxidants face challenges in penetrating the skin barrier due to their high molecular weight, which limits their transdermal delivery. While some nanoformulations have improved penetration, their effectiveness varies based on particle size, carrier type and formulation technique.

#### Limited skin absorption of high‐molecular weight antioxidants

*Tinospora cordifolia* nanoparticles successfully enhanced bioavailability, but many plant extracts still lack effective penetration systems [81]. High molecular weight polysaccharides from *Diospyros kaki* (DK‐H) and brown algae‐derived extracts exhibited low dermal absorption, necessitating carrier modifications to enhance skin penetration [8, 10, 28, 57, 59]. In some cases, caffeic acid in lipid vesicular gels (LVGs) remained primarily within the formulation, reducing its ability to reach deeper skin layers [69].

#### Variability in Nanoformulation efficacy

Nanoformulations have shown promise in improving antioxidant delivery, but their penetration efficiency remains inconsistent. Nanoemulsions containing eugenol and limonene demonstrated variable penetration, highlighting the need for further optimization to achieve targeted skin delivery [73]. Similarly, quercetin‐loaded liposomes, chitosomes and micelles exhibited inconsistent penetration rates, requiring further refinement to optimize dermal absorption [11, 52, 53].

#### Inconsistent deposition in advanced delivery systems

While bacterial cellulose membranes infused with *Epilobium angustifolium* [57] and polyoxazoline micelles [11] have improved antioxidant delivery, other advanced nanoformulations, such as blueberry‐loaded liposomes [44] and verbascoside derivatives [65], display inconsistent skin deposition. These inconsistencies affect bioavailability, making it difficult to ensure predictable efficacy in skincare formulations.

### Standardization and reproducibility issues

Botanical antioxidants often exhibit variability due to differences in geographical origin, seasonal changes and processing techniques, leading to batch‐to‐batch inconsistencies. These variations create challenges in ensuring consistent bioactivity, efficacy and regulatory compliance for large‐scale production.

#### Variability in polyphenol content

Studies on *Malus sylvestris* and palm kernel cake (PKC) extracts have shown significant fluctuations in polyphenol concentrations, emphasizing the need for standardized extraction methods to maintain consistency in antioxidant potency [33, 58].

#### Challenges in fermented botanical extracts

Fermentation has been shown to enhance bioactivity in botanical extracts, but differences in microbial metabolism lead to inconsistencies in their final composition. Variability in fermentation conditions makes quality control challenging, affecting reproducibility in cosmeceutical formulations [13].

#### Geographical influence on honey‐based formulations

Honey‐based formulations also exhibit significant phytochemical differences depending on their geographical origin. These variations complicate large‐scale standardization, as the antioxidant and antibacterial properties of honey can differ based on environmental factors, floral sources and regional climate conditions [31].

### High cost and commercial viability concerns

The development of advanced antioxidant formulations, such as nanoemulsions, lipid nanocapsules (LNCs) and magnetoliposomes, enhances bioavailability and skin penetration. However, high manufacturing costs pose a significant barrier to their mass‐market adoption, limiting their accessibility in commercial skincare products.

#### Cost barriers in advanced formulations

While nanoencapsulation technologies improve bioactive stability and delivery, their production remains expensive, reducing their feasibility for low‐cost skincare applications. High costs associated with specialized materials, equipment and formulation processes make large‐scale implementation challenging [6, 53, 54, 73].

#### Sustainability and cost‐effectiveness in extraction methods

Environmentally friendly extraction methods, such as deep eutectic solvent (NaDES)‐based grape pomace extractions, offer a sustainable alternative to traditional solvent‐based methods. However, despite their eco‐friendly nature, these extraction techniques require cost‐effective modifications to ensure scalability and affordability in commercial applications [6].

Table 8 outlines critical challenges in stabilizing antioxidant formulations. Photostability issues, such as rapid degradation of EGCG, necessitate protective agents. Encapsulation leakage remains a challenge, with liposomes and niosomes experiencing up to 10% content loss over 3 months. Antioxidants like carotenoids and polyphenols are highly sensitive to oxidation, requiring protective environments to maintain efficacy. Hydrogels demand precise viscosity control (310–450 cP) to ensure user‐friendly application. Limited skin penetration of certain antioxidants, such as rutin, necessitates advanced delivery systems like ethosomes for enhanced absorption. Addressing these formulation barriers is crucial for maintaining potency, longevity and consumer satisfaction in antioxidant skincare.

**TABLE 8:** Formulation challenges and stability concerns.

Challenge	Impact on formulations	References
Photostability issues	EGCG (epigallocatechin gallate) degraded rapidly under UV exposure; alpha‐lipoic acid improved stability	[86]
Encapsulation leakage	Niosomes and liposomes exhibited 5–10% leakage over 3 months, requiring improved stabilizers	[52, 74]
Oxidation sensitivity	Carotenoids and polyphenols oxidized within 4 weeks in light‐exposed conditions without protection	[46, 79]
Viscosity control	Hydrogels required optimal polymer ratio (310–450 cP) for spreadability and user acceptance	[8, 40, 55, 62]
Skin penetration limitation	Free rutin showed low epidermal penetration, requiring ethosome‐based delivery for deeper absorption	[9]

Table 9 presents regulatory challenges in ensuring antioxidant formulations meet safety and compliance standards. Cytotoxicity assessments confirm that tested formulations remain within safe limits (<20% for HaCaT keratinocytes). Allergenic risks from essential oils necessitate low concentrations (<1%) to prevent skin irritation. Heavy metal contamination testing via ICP‐MS ensures botanical extracts meet FDA safety limits. Microbial contamination prevention is achieved through stability testing, with preservative systems effectively maintaining sterility for 6 months. These regulatory considerations underscore the importance of rigorous safety evaluations, ensuring consumer‐friendly, non‐toxic and compliant skincare formulations.

**TABLE 9:** Regulatory and safety considerations in skincare applications.

Regulatory concern	Key observations and compliance considerations	References
Cytotoxicity limits	Cytotoxicity threshold maintained at <20% for HaCaT keratinocytes in tested formulations	[13, 44, 57, 81]
Allergenicity and skin sensitization	Essential oils (basil, oregano) required low concentrations (<1%) to prevent irritation	[8]
Heavy metal contamination	Botanical extracts analysed via ICP‐MS met FDA limits for arsenic, lead and cadmium (<0.5 ppm)	[21, 45]
Microbial contamination prevention	Stability testing confirmed the absence of bacterial growth for 6 months with natural preservative systems	[31, 32, 36]

## FUTURE DIRECTIONS IN NATURAL‐BASED ANTIOXIDANT RESEARCH

Despite significant advancements in anti‐ageing, photoprotection and anti‐inflammatory skincare, key challenges remain in bioavailability, formulation stability, regulatory validation and large‐scale dermatological adoption. To fully harness the potential of botanical antioxidants, future research must focus on enhanced delivery systems, large‐scale human trials, sustainable extraction techniques, microbiome interactions and regulatory compliance.

### Enhancing bioavailability with advanced delivery systems

A major limitation of botanical antioxidants is their poor skin penetration, primarily due to their molecular size and hydrophilicity. Nanotechnology‐based formulations such as nanoemulsions, microneedles and lipid‐based carriers could significantly improve antioxidant delivery into deeper skin layers [1, 8, 59, 68, 82]. Additionally, integrating bacterial cellulose membranes into sustained‐release patches may offer long‐term antioxidant delivery, enhancing prolonged skincare benefits [57]. Hybrid nanocarriers—including liposomal, ethosomal and niosomal systems—have shown promise in improving penetration and bioavailability [26, 70, 74]. Similarly, layer‐by‐layer coatings in oil‐in‐water emulsions could enhance antioxidant stability, ensuring long‐lasting activity in cosmeceutical formulations [78]. Hybrid nanoemulsions combining multiple bioactives may also provide synergistic antioxidant and UV‐protective effects, improving photostability and sunscreen efficacy [41, 115].

### Developing multi‐targeted skincare formulations

To enhance hydration, collagen production and skin barrier repair, botanical antioxidants should be combined with peptides, ceramides and hydrating agents. Future formulations should explore synergistic blends of antioxidants with hyaluronic acid and ceramides to optimize anti‐ageing and hydration benefits [59, 66]. Additionally, marine algae, cyanobacteria and fungal‐derived antioxidants could complement ceramides and plant sterols, further improving skin elasticity and hydration [16, 28, 31, 34]. Botanical antioxidants also exhibit natural UV‐absorbing properties and could be combined with synthetic UV filters to develop broad‐spectrum, long‐lasting sunscreen formulations. Future research should focus on hybrid sun‐care products that integrate natural SPF agents with synthetic UV filters for enhanced UV protection and photostability [23, 50, 62, 84].

### Expanding Large‐Scale Human Clinical Trials & Regulatory Approvals

Despite promising preclinical and small‐scale human trials, large‐scale, placebo‐controlled clinical studies are necessary to establish long‐term safety, efficacy and dosage standardization. More randomized human trials are needed to validate dermatological benefits, particularly for chronic skin conditions such as psoriasis, eczema and acne scars [1, 4, 35, 85]. To ensure widespread adoption, botanical‐based cosmeceuticals must align with international regulatory standards. Regulatory approval pathways such as FDA GRAS (Generally Recognized as Safe), EMA botanical monographs and COSMOS certification should be pursued to standardize botanical‐derived formulations and facilitate global market acceptance [16, 35, 36, 85].

### Sustainable & Green Extraction Optimization

Eco‐friendly extraction methods must be prioritized to reduce solvent use, maximize bioactive yield and enhance sustainability. Technologies such as supercritical CO_2_ and ultrasound‐assisted extraction should be further optimized to increase efficiency while minimizing environmental impact [18, 33, 37, 58, 64]. Additionally, microwave‐assisted extractions should be explored for their ability to preserve antioxidant activity and increase bioavailability [91]. A circular economic approach should also be adopted by transforming agricultural waste into high‐value cosmeceuticals, promoting sustainability and cost‐effectiveness. Upcycling plant‐based waste materials such as palm kernel cake, grape peel and dragon fruit peel could provide environmentally friendly and scalable skincare solutions [23, 33, 68]. Similarly, coffee silverskin and chestnut inner shell extracts should be investigated as alternative antioxidant sources in dermatological formulations [12, 32].

### Exploring skin microbiome‐antioxidant interaction

The relationship between botanical antioxidants and skin microbiomes remains underexplored. Investigating how antioxidants influence microbial diversity and inflammation could lead to the development of next‐generation probiotic and prebiotic skincare formulations that support microbial balance and skin health [5, 7, 31, 35, 75, 83]. Future research should focus on understanding how botanical extracts interact with skin microbiota to enhance personalized skincare targeting inflammation, ageing and barrier repair. By integrating microbiome‐friendly antioxidants, new cosmeceuticals could be developed to prevent dysbiosis while promoting healthier skin [38].

### Developing photostable and pro‐oxidant‐free antioxidants

Some botanical antioxidants can function as pro‐oxidants under UV exposure, leading to oxidative stress instead of protection. Future research should focus on screening antioxidants for photosensitization risks to prevent unintended skin damage [49]. To improve formulation stability, combining antioxidants with stabilizers such as alpha‐lipoic acid and vitamin C should be optimized for UV‐exposed skincare formulations [86].

Future advancements in delivery systems, sustainable extraction, microbiome research and regulatory compliance will be crucial in overcoming the current challenges faced by botanical antioxidants. By integrating eco‐friendly practices, advanced nanotechnology and clinical validation, natural antioxidants can become more effective, stable and accessible for dermatological and cosmeceutical applications.

Table 10 showcases how combining antioxidants enhances their protective, anti‐inflammatory and photoprotective effects. Resveratrol with niacinamide and GHK peptide mitigates oxidative stress and promotes skin defence via NRF2 pathways. Green tea polyphenols with CoQ10 accelerate skin barrier recovery, reducing inflammation. Essential oil combinations, such as calendula with geranium, improve UV protection and stability. Propolis with polyphenols provides broad‐spectrum UV defence while preventing lipid peroxidation. The data support the trend of synergistic antioxidant formulations, where well‐selected combinations enhance efficacy, prolong skin benefits and improve formulation stability.

**TABLE 10:** Synergistic and combined effects of antioxidants in skincare.

Combination	Observed synergistic effect	References
Resveratrol + niacinamide + GHK peptide	Reduced PM2.5‐induced oxidative stress, downregulated IL‐6 and CXCL10, upregulated NRF2 protective pathways	[90]
Green tea polyphenols + Coenzyme Q10 + ascorbyl tetraisopalmitate	Accelerated skin barrier recovery, minimized SDS‐induced inflammation, reduced transepidermal water loss	[75]
*Calendula officinalis* + geranium essential oil	Provided broad‐spectrum SPF protection, enhanced photostability and prolonged UV protection	[50]
Hesperetin + ferulic acid + menthol essential oil	Sustained drug release for 42.63% over 144 h, improved skin hydration, increased anti‐inflammatory action	[47]
Propolis + polyphenols	Broad UV protection, inhibited lipid peroxidation and reduced oxidative stress in fibroblasts	[36]

Table 11 highlights key trends shaping the future of antioxidant‐based skincare. Sustainable extraction methods, such as supercritical CO_2_ and ultrasound‐assisted techniques, reduce solvent waste by 60%, promoting eco‐friendly production. Biodegradable nanocarriers like polyoxazoline (POx)‐based systems offer higher antioxidant loading and enhanced penetration. Fermentation techniques improve phenolic content by 40%, boosting bioavailability. Advances in DNA‐repair‐boosting antioxidants show promise in preventing UV‐induced genetic damage. The hybridization of natural antioxidants with synthetic stabilizers extends shelf‐life and enhances performance. These trends emphasize the industry's shift towards sustainability, biocompatibility and high‐efficacy formulations to meet evolving consumer and environmental demands.

**TABLE 11:** Emerging trends and future directions in antioxidant skincare.

Trend	Recent developments and future implications	References
Sustainable extraction methods	Supercritical CO_2_ and ultrasound‐assisted extraction reduced solvent use by 60%, improving sustainability	[27, 37, 64]
Biodegradable nanocarriers	Polyoxazoline (POx)‐based lipid nanocapsules showed 3× higher antioxidant loading vs. PEG‐based carriers	[54]
Probiotic‐fermented antioxidants	Fermentation enhanced phenolic content by 40%, improving bioavailability and skincare benefits	[12, 60]
DNA repair‐boosting antioxidants	Antioxidant complexes improved UVB‐induced DNA repair, reducing ROS‐mediated genetic damage	[30, 75, 77]
Hybrid natural‐synthetic formulations	Synergistic combinations of natural flavonoids with synthetic stabilizers prolonged antioxidant stability	[15, 25, 70]

## CONCLUSION

Natural‐based antioxidants, sourced from plants, algae, fungi and bee products, represent a scientifically validated and sustainable approach to anti‐ageing skincare. Their ability to neutralize oxidative stress, inhibit collagen‐degrading enzymes, promote skin hydration and provide UV protection makes them invaluable in cosmeceutical and dermatological applications. The advancement of sustainable extraction techniques, including NaDES, UAE and fermentation‐assisted methods, has significantly enhanced bioactivity, environmental sustainability and resource efficiency. Furthermore, innovations in nanoencapsulation and delivery systems—such as nanoemulsions, liposomes, magnetoliposomes and chitosomes—have improved antioxidant bioavailability, controlled release and dermal penetration, addressing long‐standing formulation challenges.

However, limitations remain, including photostability concerns, bioavailability variability, high production costs and regulatory hurdles. Future research should focus on synergistic formulations, integrating antioxidants with peptides, ceramides and synthetic UV filters to optimize anti‐ageing efficacy and photoprotection. Additionally, large‐scale clinical trials are essential for validating dermatological benefits and securing regulatory approvals for mainstream adoption.

Sustainability remains at the forefront of cosmeceutical innovation. The upcycling of agricultural and marine by‐products, such as grape pomace, coffee silverskin and red algae extracts, aligns with the principles of a circular economy, reinforcing the demand for eco‐friendly, high‐performance skincare solutions. As research advances, integrating microbiome‐friendly antioxidants and hybrid nanocarrier technologies will further refine the efficacy and market viability of these bioactives.

By leveraging cutting‐edge scientific advancements and sustainability‐driven formulations, natural‐based antioxidants are poised to redefine anti‐ageing skincare. Their multifunctional properties, enhanced bioengineering and commitment to eco‐conscious innovation position them as key drivers in the evolution of next‐generation cosmeceuticals.

## CONFLICT OF INTEREST STATEMENT

The authors declare that they have no conflict of interest.

## DISCLOSURE

Authors partly used OpenAI Large‐Scale Language Model to maximize accuracy, clarity and organization.

## Data Availability

The data that support the findings of this study are available from the corresponding author upon reasonable request.
